# Supplementary material for: Seed encrusting with salicylic acid: A novel approach to improve establishment of grass species in ecological restoration
Source: PLoS One. 2021 Jun 9;16(6):e0242035. doi: 10.1371/journal.pone.0242035 (PMC8189473; doi:10.1371/journal.pone.0242035)
Supplement: S1 File — (PDF) [file pone.0242035.s001.pdf]

**LINE 1**	RC	AIS	AEN	REN	MES	AC	MIS	MC	MEN	AES	RES	AIN	RIN	MIN	RIS

**LINE2**	AC	RES	RIS	RC	AES	REN	RIN	MIN	MC	MES	AIN	MIS	MEN	AIS	AEN

**LINE 3**	MIN	MEN	MES	AES	MIS	AIN	AC	RIS	RES	RC	MC	RIN	AEN	REN	AIS

**LINE 4**	AIS	MEN	RIN	MC	AES	RES	RIS	MIN	RC	MIS	AC	AEN	AIN	MES	REN

DATALOG

BAGS 1

BAGS 2

BAGS 3

BAGS 4

BOX 1

AC	REN	AEN
MC	MIS	MEN
AIS	AES	RC
MES	MIN	AIN
RIS	RES	RIN

BOX 4

AES	MIN	MES
AEN	MEN	RIS
AIS	AC	RC
RES	MC	REN
MIS	RIN	AIN

BOX 2

AC	AEN	RES
REN	RIS	AES
MEN	RIN	AIS
RC	MIS	AIN
MC	MES	MIN

BOX 3

AIN	AC	MIS
RC	AIS	REN
RIS	MEN	MIN
RES	RIN	MC
AEN	MES	AES

AC	Austrostipa control
AIN	Austrostipa imbibed
AIS	Austrostipa imbibed with SA
AEN	Austrostipa encrusted
AES	Austrostipa encrusted with SA
MC	Micolaena control
MIN	Micolaena imbibed
MIS	Micolaena imbibed with SA
MEN	Micolaena encrusted
MES	Micolaena encrusted with SA
RC	Ryditosperma control
RIN	Ryditosperma imbibed
RIS	Ryditosperma imbibed with SA
REN	Ryditosperma encrusted
RES	Ryditosperma encrusted with SA

Fence
