# Supplementary material for: Seed encrusting with salicylic acid: A novel approach to improve establishment of grass species in ecological restoration
Source: PLoS One. 2021 Jun 9;16(6):e0242035. doi: 10.1371/journal.pone.0242035 (PMC8189473; doi:10.1371/journal.pone.0242035)

Soil Moisture Retention Curve (SMRC) with the Dew Point potentiometer WP4C

Sample	grav %1	Volumetric	Mpa
a	0.314	0.430	0.09
a	0.255	0.350	0.13
a	0.132	0.180	0.26
a	0.142	0.194	0.32
a	0.088	0.121	0.9
a	0.053	0.072	11.74
b	0.267	0.366	0.13
b	0.206	0.283	0.2
b	0.145	0.199	0.22
b	0.082	0.112	0.65
b	0.042	0.057	14.78
c	0.347	0.475	0.17
c	0.288	0.394	0.2
c	0.229	0.313	0.23
c	0.165	0.227	0.18
c	0.089	0.122	1.16
c	0.054	0.074	30.26

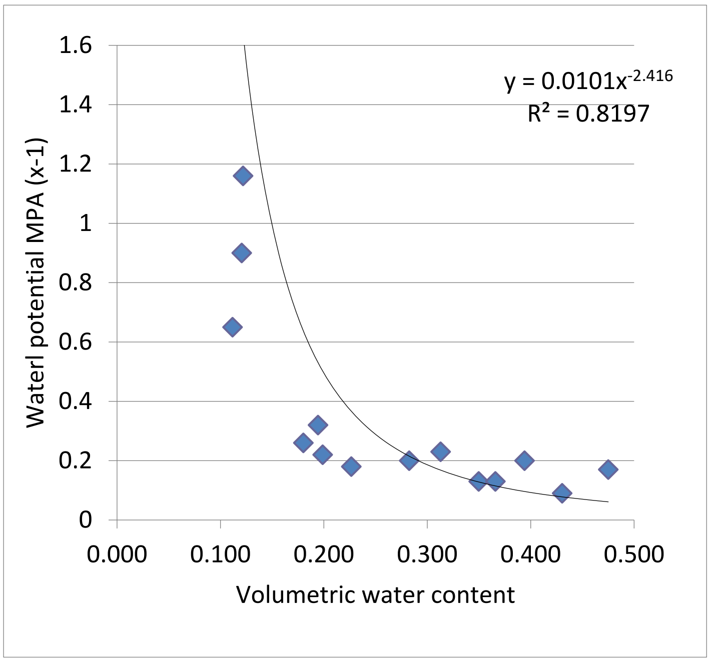

1.Mix soil with water to “field capacity” (i.e. with as much water as you can).
2.Measure weight of a clean steel cup before placing soil sample on it.
3.Place soil sample on steel cup.
4.Measure water potential (in WP4C).
5.Measure weight of the wet soil and sample cup.
6.Dry soil sample in the oven (10 minutes at 75 ºC) and repeat until reaches below -10 MPa
7.Dry soil sample at 105 ºC for 24h or until reaching a constant weight (which indicates that the soil is dry).
7.Measure the weight of the dry soil and sample cup to estimate the soil weight and soil water content for the previous measurements.
8.Estimate gravimetric water content % (gr_{water}/gr_{soil}).
9.Measure density of the soil.
10.Transform gravimetric water content to volumetric water content. The simple way to calculate volumetric water content is multiplying gravimetric by soil density in gr/cm^3.

Average weekly volumetric soil water content recorded

1	0.087157
2	0.161447
3	0.152323
4	0.139742
5	0.113782
6	0.111499
7	0.133981
8	0.134733
9	0.155279
10	0.159133
11	0.168772
12	0.167996

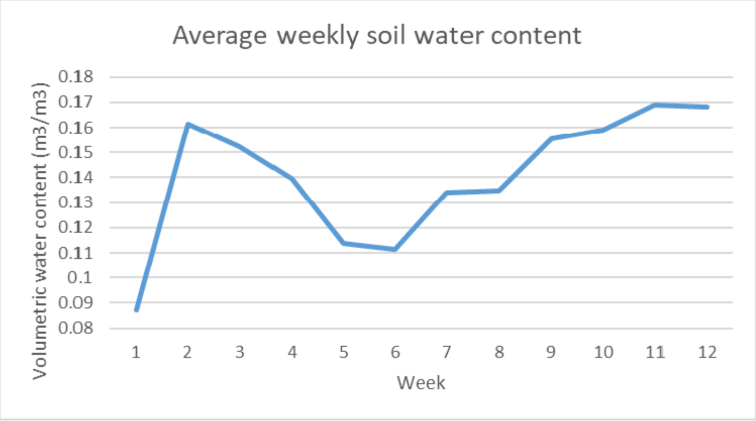

Supplement: S2 File — (PDF) [file pone.0242035.s002.pdf]
