# Supplementary material for: Seed encrusting with salicylic acid: A novel approach to improve establishment of grass species in ecological restoration
Source: PLoS One. 2021 Jun 9;16(6):e0242035. doi: 10.1371/journal.pone.0242035 (PMC8189473; doi:10.1371/journal.pone.0242035)
Supplement: S3 File — Summary tables are provided for each species with the averages and standard errors for mortality, germination, and germination adjusted for viability. (PDF) [file pone.0242035.s003.pdf]

Austrostipa scabra laboratory germiation experiment

Code	Sp	Trt	Rep	WP	Tot	2	3	4	5	7	10	15	21	left	dead	totF
AC	Aus	ctrl	1	0	25	0	3	16	17	18	18	18	18	7	3	22
AC	Aus	ctrl	2	0	25	2	5	14	16	18	18	21	21	4	3	22
AC	Aus	ctrl	3	0	25	1	4	11	13	16	16	16	16	9	4	21
AC	Aus	ctrl	4	0	25	2	6	14	15	17	17	17	17	8	6	19
AIN	Aus	in	1	0	25	1	10	14	15	15	16	16	16	9	6	19
AIN	Aus	in	2	0	25	3	10	19	21	22	23	23	23	2	0	25
AIN	Aus	in	3	0	25	3	10	14	15	17	17	17	17	8	4	21
AIN	Aus	in	4	0	25	2	9	17	18	21	21	21	21	4	4	21
AIS	Aus	is	1	0	25	3	13	18	19	21	21	21	21	4	2	23
AIS	Aus	is	2	0	25	4	9	14	15	17	17	17	17	8	4	21
AIS	Aus	is	3	0	25	4	7	13	14	15	15	16	16	9	6	19
AIS	Aus	is	4	0	25	3	9	15	16	16	17	17	17	8	5	20
AEN	Aus	en	1	0	25	2	8	13	15	17	17	17	17	8	4	21
AEN	Aus	en	2	0	25	3	9	18	19	19	19	19	19	6	4	21
AEN	Aus	en	3	0	25	4	11	16	18	19	20	20	20	5	3	22
AEN	Aus	en	4	0	25	3	10	18	19	21	21	21	21	4	3	22
AES	Aus	es	1	0	25	3	10	13	13	13	13	14	14	11	6	19
AES	Aus	es	2	0	25	3	13	19	19	20	20	20	20	5	2	23
AES	Aus	es	3	0	25	2	9	18	23	23	23	23	23	2	1	24
AES	Aus	es	4	0	25	2	11	15	16	17	18	18	18	7	2	23
AC	Aus	ctrl	1	-0.6	25	0	4	13	16	17	18	19	19	6	3	22
AC	Aus	ctrl	2	-0.6	25	0	5	12	13	15	15	15	15	10	6	19
AC	Aus	ctrl	3	-0.6	25	0	5	8	9	10	11	11	11	14	10	15
AC	Aus	ctrl	4	-0.6	25	0	1	7	10	12	13	13	13	12	5	20
AIN	Aus	in	1	-0.6	25	0	7	20	20	20	20	21	21	4	1	24
AIN	Aus	in	2	-0.6	25	0	5	14	17	19	19	19	19	6	4	21
AIN	Aus	in	3	-0.6	25	0	5	14	16	17	17	17	17	8	5	20
AIN	Aus	in	4	-0.6	25	0	4	13	16	17	18	19	19	6	2	23
AIS	Aus	is	1	-0.6	25	0	5	14	17	17	17	18	18	7	4	21
AIS	Aus	is	2	-0.6	25	0	5	13	13	13	14	14	14	11	11	14
AIS	Aus	is	3	-0.6	25	0	8	18	19	20	21	21	21	4	3	22
AIS	Aus	is	4	-0.6	25	0	6	17	19	19	19	19	19	6	4	21
AEN	Aus	en	1	-0.6	25	0	5	12	17	18	18	19	19	6	4	21
AEN	Aus	en	2	-0.6	25	0	5	11	14	15	16	17	17	8	4	21
AEN	Aus	en	3	-0.6	25	0	7	15	16	18	18	18	18	7	1	24
AEN	Aus	en	4	-0.6	25	0	7	13	17	17	17	18	18	7	3	22
AES	Aus	es	1	-0.6	25	0	4	9	13	16	18	19	19	6	2	23
AES	Aus	es	2	-0.6	25	0	4	12	14	16	16	17	17	8	5	20
AES	Aus	es	3	-0.6	25	0	5	13	16	17	17	17	17	8	4	21
AES	Aus	es	4	-0.6	25	0	5	11	14	17	17	17	17	8	3	22
AC	Aus	ctrl	1	-0.9	25	0	1	1	7	8	9	10	10	15	5	20
AC	Aus	ctrl	2	-0.9	25	0	2	2	5	8	12	13	14	11	6	19
AC	Aus	ctrl	3	-0.9	25	0	2	2	8	13	16	16	17	8	3	22
AC	Aus	ctrl	4	-0.9	25	0	3	3	9	12	15	16	16	9	6	19
AIN	Aus	in	1	-0.9	25	0	2	8	13	15	16	16	16	9	7	18
AIN	Aus	in	2	-0.9	25	0	3	8	12	13	13	15	15	10	5	20
AIN	Aus	in	3	-0.9	25	0	4	11	12	14	16	16	16	9	6	19
AIN	Aus	in	4	-0.9	25	0	2	7	12	14	15	16	17	8	3	22

Code	Sp	Trt	Rep	WP	Tot	2	3	4	5	7	10	15	21	left	dead	totF
AIS	Aus	is	1	-0.9	25	0	1	6	10	13	13	13	13	12	8	17
AIS	Aus	is	2	-0.9	25	0	3	9	12	14	16	16	16	9	4	21
AIS	Aus	is	3	-0.9	25	0	4	8	13	17	17	17	17	8	1	24
AIS	Aus	is	4	-0.9	25	0	5	12	17	21	22	22	24	1	9	16
AEN	Aus	en	1	-0.9	25	0	3	10	14	15	16	17	17	8	3	22
AEN	Aus	en	2	-0.9	25	0	4	9	14	19	19	19	19	6	3	22
AEN	Aus	en	3	-0.9	25	0	3	8	13	15	16	17	17	8	5	20
AEN	Aus	en	4	-0.9	25	0	1	5	8	11	12	12	12	13	9	16
AES	Aus	es	1	-0.9	25	0	3	5	9	13	13	15	15	10	5	20
AES	Aus	es	2	-0.9	25	0	1	3	12	17	19	20	20	5	2	23
AES	Aus	es	3	-0.9	25	0	2	7	12	15	16	17	17	8	4	21
AES	Aus	es	4	-0.9	25	0	3	6	8	14	14	14	14	11	9	16
AC	Aus	ctrl	1	-1.2	25	0	0	0	1	4	6	9	10	15	6	19
AC	Aus	ctrl	2	-1.2	25	0	0	0	1	5	6	9	9	16	4	21
AC	Aus	ctrl	3	-1.2	25	0	0	0	2	6	6	9	11	14	4	21
AC	Aus	ctrl	4	-1.2	25	0	0	0	1	4	4	8	9	16	6	19
AIN	Aus	in	1	-1.2	25	0	0	0	1	4	5	8	8	17	5	20
AIN	Aus	in	2	-1.2	25	0	0	0	2	6	10	12	12	13	5	20
AIN	Aus	in	3	-1.2	25	0	0	0	2	8	10	11	11	14	8	17
AIN	Aus	in	4	-1.2	25	0	0	0	0	5	7	8	8	17	8	17
AIS	Aus	is	1	-1.2	25	0	0	0	6	12	12	14	14	11	5	20
AIS	Aus	is	2	-1.2	25	0	0	0	3	9	10	11	11	14	8	17
AIS	Aus	is	3	-1.2	25	0	0	0	2	5	8	11	11	14	6	19
AIS	Aus	is	4	-1.2	25	0	0	0	0	6	8	8	8	17	10	15
AEN	Aus	en	1	-1.2	25	0	0	0	4	9	12	13	13	12	4	21
AEN	Aus	en	2	-1.2	25	0	0	0	2	5	9	11	11	14	8	17
AEN	Aus	en	3	-1.2	25	0	0	0	2	6	10	12	12	13	7	18
AEN	Aus	en	4	-1.2	25	0	0	0	1	3	5	9	9	16	7	18
AES	Aus	es	1	-1.2	25	0	0	0	4	5	6	9	9	16	6	19
AES	Aus	es	2	-1.2	25	0	0	0	2	6	8	10	10	15	7	18
AES	Aus	es	3	-1.2	25	0	0	0	3	7	10	12	12	13	5	20
AES	Aus	es	4	-1.2	25	0	0	0	3	7	10	13	13	12	6	19

ctrl: untreated control
en: encrusted witouh SA
es: encrusted with SA
in: imbibed witouh SA
is: imbibved with SA

Summary *Austrostipa scabra* laboratory germiation experiment

WP	Code	Avg D	St.err D	Avg Gt	St.err Gt	Avg G adj	St.err G adj
0	AC	16.00%	2.45%	72.00%	3.74%	85.73%	3.66%
	AEN	14.00%	1.00%	77.00%	2.96%	89.45%	2.64%
	AES	11.00%	3.84%	75.00%	6.54%	83.68%	4.24%
	AIN	14.00%	4.36%	77.00%	5.72%	89.29%	3.69%
	AIS	17.00%	2.96%	71.00%	3.84%	85.37%	1.87%
-0.6	AC	24.00%	5.10%	58.00%	5.92%	75.91%	3.91%
	AEN	12.00%	2.45%	72.00%	1.41%	82.06%	2.76%
	AES	14.00%	2.24%	70.00%	1.73%	81.46%	1.41%
	AIN	12.00%	3.16%	76.00%	2.83%	86.40%	1.46%
	AIS	22.00%	6.40%	72.00%	5.10%	92.91%	2.67%
-0.9	AC	20.00%	2.45%	57.00%	5.36%	71.29%	6.43%
	AEN	20.00%	4.90%	65.00%	5.17%	80.91%	2.43%
	AES	20.00%	5.10%	66.00%	4.58%	82.60%	2.54%
	AIN	21.00%	2.96%	64.00%	1.41%	81.34%	2.76%
	AIS	22.00%	6.40%	70.00%	8.06%	93.37%	16.39%
-1.2	AC	20.00%	2.00%	39.00%	1.66%	48.81%	2.01%
	AEN	26.00%	3.00%	45.00%	2.96%	60.82%	3.24%
	AES	24.00%	1.41%	44.00%	3.16%	57.84%	3.80%
	AIN	26.00%	3.00%	39.00%	3.57%	52.94%	4.94%
	AIS	29.00%	3.84%	44.00%	4.24%	61.48%	3.18%

Avg D: average mortality
St.err D: standard error mortality

Avg Gt: average germination (keeping dead seeds in the total)
St.err D: standard error germination (keeping dead seeds in the total)

Avg G adj: average germination adjusted from viability (removing dead seeds from the total)
St.err G adj: standard error germination adjusted from viability (removing dead seeds from the total)

Microlaena stipoides laboratory germiation experiment

Code	Sp	Trt	Rep	WP	Tot	2	3	4	5	7	10	15	21	left	dead	totF
MC	Mic	ctrl	1	0	25	2	10	15	17	17	17	17	17	8	3	22
MC	Mic	ctrl	2	0	25	2	8	13	14	14	16	16	16	9	4	21
MC	Mic	ctrl	3	0	25	2	9	13	15	16	17	17	17	8	5	20
MC	Mic	ctrl	4	0	25	3	6	11	13	13	13	13	13	12	2	23
MIN	Mic	in	1	0	25	1	10	14	15	17	18	18	18	7	4	21
MIN	Mic	in	2	0	25	3	9	13	13	14	14	14	14	11	3	22
MIN	Mic	in	3	0	25	0	4	9	9	10	11	11	11	14	3	22
MIN	Mic	in	4	0	25	3	7	9	10	12	13	14	14	11	3	22
MIS	Mic	is	1	0	25	1	6	8	10	11	13	13	13	12	2	23
MIS	Mic	is	2	0	25	3	9	13	13	15	15	15	15	10	2	23
MIS	Mic	is	3	0	25	2	9	10	13	13	13	13	13	12	0	25
MIS	Mic	is	4	0	25	2	6	11	12	12	12	13	13	12	3	22
MEN	Mic	en	1	0	25	6	16	20	20	20	20	22	22	3	1	24
MEN	Mic	en	2	0	25	4	14	19	19	20	21	21	21	4	2	23
MEN	Mic	en	3	0	25	3	11	14	16	17	18	20	20	5	3	22
MEN	Mic	en	4	0	25	4	16	19	19	19	19	20	20	5	1	24
MES	Mic	es	1	0	25	4	7	13	14	16	16	16	16	9	3	22
MES	Mic	es	2	0	25	4	11	13	13	13	13	14	14	11	5	20
MES	Mic	es	3	0	25	5	12	17	20	20	20	21	21	4	2	23
MES	Mic	es	4	0	25	5	15	15	16	16	16	18	18	7	3	22
MC	Mic	ctrl	1	-0.6	25	0	8	11	14	16	18	20	20	5	2	23
MC	Mic	ctrl	2	-0.6	25	0	6	12	16	19	19	20	20	5	2	23
MC	Mic	ctrl	3	-0.6	25	0	5	11	13	13	14	15	16	9	3	22
MC	Mic	ctrl	4	-0.6	25	0	6	12	12	13	13	13	13	12	6	19
MIN	Mic	in	1	-0.6	25	0	6	11	11	11	11	12	12	13	8	17
MIN	Mic	in	2	-0.6	25	0	4	11	14	15	17	20	20	5	2	23
MIN	Mic	in	3	-0.6	25	0	5	9	11	12	15	17	17	8	4	21
MIN	Mic	in	4	-0.6	25	0	5	10	11	11	13	13	13	12	10	15
MIS	Mic	is	1	-0.6	25	0	0	8	12	14	15	15	15	10	9	16
MIS	Mic	is	2	-0.6	25	0	5	9	11	12	13	14	14	11	9	16
MIS	Mic	is	3	-0.6	25	0	5	7	11	11	11	13	13	12	4	21
MIS	Mic	is	4	-0.6	25	0	5	12	15	16	16	16	16	9	6	19
MEN	Mic	en	1	-0.6	25	0	7	13	14	16	16	17	17	8	4	21
MEN	Mic	en	2	-0.6	25	0	9	16	17	17	17	20	21	4	2	23
MEN	Mic	en	3	-0.6	25	0	5	14	17	20	20	21	21	4	2	23
MEN	Mic	en	4	-0.6	25	0	12	14	14	14	14	16	17	8	3	22
MES	Mic	es	1	-0.6	25	0	8	15	15	16	16	17	17	8	6	19
MES	Mic	es	2	-0.6	25	0	7	11	13	17	18	19	19	6	6	19
MES	Mic	es	3	-0.6	25	0	8	14	14	15	16	16	16	9	8	17
MES	Mic	es	4	-0.6	25	0	6	10	13	15	15	15	15	10	9	16
MC	Mic	ctrl	1	-0.9	25	0	0	3	11	13	16	16	16	9	5	20
MC	Mic	ctrl	2	-0.9	25	0	0	6	14	15	16	16	16	9	6	19
MC	Mic	ctrl	3	-0.9	25	0	0	5	11	13	14	15	16	9	4	21
MC	Mic	ctrl	4	-0.9	25	0	0	5	9	11	12	14	14	11	7	18
MIN	Mic	in	1	-0.9	25	0	0	6	13	14	15	16	16	9	4	21
MIN	Mic	in	2	-0.9	25	0	0	5	9	10	13	14	14	11	6	19
MIN	Mic	in	3	-0.9	25	0	0	4	9	13	13	14	15	10	5	20
MIN	Mic	in	4	-0.9	25	0	0	3	8	13	14	15	15	10	5	20

Code	Sp	Trt	Rep	WP	Tot	2	3	4	5	7	10	15	21	left	dead	totF
MIS	Mic	is	1	-0.9	25	0	0	4	9	10	11	11	13	12	2	23
MIS	Mic	is	2	-0.9	25	0	0	7	11	13	13	13	13	12	6	19
MIS	Mic	is	3	-0.9	25	0	0	5	13	14	14	15	16	9	6	19
MIS	Mic	is	4	-0.9	25	0	0	7	12	12	12	15	15	10	8	17
MEN	Mic	en	1	-0.9	25	0	0	7	11	14	14	15	15	10	9	16
MEN	Mic	en	2	-0.9	25	0	0	9	14	18	19	21	21	4	3	22
MEN	Mic	en	3	-0.9	25	0	0	7	13	15	16	16	16	9	6	19
MEN	Mic	en	4	-0.9	25	0	0	11	16	17	18	18	18	7	3	22
MES	Mic	es	1	-0.9	25	0	0	8	14	15	16	16	16	9	6	19
MES	Mic	es	2	-0.9	25	0	0	8	12	15	16	17	17	8	5	20
MES	Mic	es	3	-0.9	25	0	0	5	13	16	16	19	19	6	2	23
MES	Mic	es	4	-0.9	25	0	0	7	11	14	14	15	15	10	6	19
MC	Mic	ctrl	1	-1.2	25	0	0	1	4	6	9	12	12	13	5	20
MC	Mic	ctrl	2	-1.2	25	0	0	0	4	6	9	10	10	15	8	17
MC	Mic	ctrl	3	-1.2	25	0	0	0	3	7	12	15	15	10	8	17
MC	Mic	ctrl	4	-1.2	25	0	0	0	4	6	8	12	12	13	3	22
MIN	Mic	in	1	-1.2	25	0	0	0	4	7	10	13	13	12	7	18
MIN	Mic	in	2	-1.2	25	0	0	0	3	5	6	9	10	15	5	20
MIN	Mic	in	3	-1.2	25	0	0	0	3	6	11	13	13	12	3	22
MIN	Mic	in	4	-1.2	25	0	0	0	4	6	9	12	12	13	6	19
MIS	Mic	is	1	-1.2	25	0	0	0	4	9	10	11	11	14	6	19
MIS	Mic	is	2	-1.2	25	0	0	0	3	7	9	10	10	15	5	20
MIS	Mic	is	3	-1.2	25	0	0	0	2	4	5	7	7	18	7	18
MIS	Mic	is	4	-1.2	25	0	0	0	4	9	12	12	14	11	1	24
MEN	Mic	en	1	-1.2	25	0	0	0	4	7	10	13	13	12	5	20
MEN	Mic	en	2	-1.2	25	0	0	0	4	8	16	16	17	8	4	21
MEN	Mic	en	3	-1.2	25	0	0	0	5	9	16	18	18	7	2	23
MEN	Mic	en	4	-1.2	25	0	0	0	8	16	19	19	19	6	1	24
MES	Mic	es	1	-1.2	25	0	0	0	6	11	15	15	15	10	4	21
MES	Mic	es	2	-1.2	25	0	0	0	5	9	11	11	13	12	5	20
MES	Mic	es	3	-1.2	25	0	0	0	5	11	14	18	19	6	3	22
MES	Mic	es	4	-1.2	25	0	0	0	5	6	9	11	14	11	3	22

ctrl: untreated control
en: encrusted witouh SA
es: encrusted with SA
in: imbibed witouh SA
is: imbibved with SA

Summary *Microlaena stipoides* laboratory germiation experiment

WP	Code	Avg D	St.err D	Avg Gt	St.err Gt	Avg G adj	St.err G adj
0	MC	14.00%	2.24%	63.00%	3.28%	73.75%	5.25%
	MEN	7.00%	1.66%	83.00%	1.66%	89.30%	1.73%
	MES	13.00%	2.18%	69.00%	5.17%	78.96%	4.18%
	MIN	13.00%	0.87%	57.00%	4.97%	65.75%	6.40%
	MIS	7.00%	2.18%	54.00%	1.73%	58.21%	2.39%
-0.6	MC	13.00%	3.28%	69.00%	5.89%	78.77%	4.17%
	MEN	11.00%	1.66%	76.00%	4.00%	85.21%	3.12%
	MES	29.00%	2.60%	67.00%	2.96%	94.34%	1.87%
	MIN	24.00%	6.32%	62.00%	6.40%	81.29%	3.31%
	MIS	28.00%	4.24%	58.00%	2.24%	81.84%	6.00%
-0.9	MC	22.00%	2.24%	62.00%	1.73%	79.54%	1.51%
	MEN	21.00%	4.97%	70.00%	4.58%	88.81%	2.94%
	MES	19.00%	3.28%	67.00%	2.96%	82.69%	1.16%
	MIN	20.00%	1.41%	60.00%	1.41%	74.97%	0.44%
	MIS	22.00%	4.36%	57.00%	2.60%	74.35%	6.34%
-1.2	MC	24.00%	4.24%	49.00%	3.57%	65.40%	6.67%
	MEN	12.00%	3.16%	67.00%	4.56%	75.84%	3.17%
	MES	15.00%	1.66%	61.00%	4.56%	71.61%	4.51%
	MIN	21.00%	2.96%	48.00%	2.45%	61.12%	3.99%
	MIS	19.00%	4.56%	42.00%	5.00%	51.28%	3.94%

Avg D: average mortality
St.err D: standard error mortality

Avg Gt: average germination (keeping dead seeds in the total)
St.err D: standard error germination (keeping dead seeds in the total)

Avg G adj: average germination adjusted from viability (removing dead seeds from the total)
St.err G adj: standard error germination adjusted from viability (removing dead seeds from the total)

Rytidosperma geniculatum laboratory germiation experiment

Code	Sp	Trt	Rep	WP	Tot	2	3	4	5	7	10	15	21	left	dead	totF
RC	Ryt	ctrl	1	0	25	2	6	12	15	15	15	15	15	10	0	25
RC	Ryt	ctrl	2	0	25	1	6	8	13	15	15	15	15	10	0	25
RC	Ryt	ctrl	3	0	25	2	6	10	12	13	13	13	13	12	3	22
RC	Ryt	ctrl	4	0	25	4	6	8	13	13	13	13	13	12	1	24
RIN	Ryt	in	1	0	25	2	6	11	12	12	13	13	13	12	1	24
RIN	Ryt	in	2	0	25	0	4	8	10	10	10	10	10	15	1	24
RIN	Ryt	in	3	0	25	1	5	8	9	10	10	10	10	15	3	22
RIN	Ryt	in	4	0	25	4	8	12	13	13	13	13	13	12	2	23
RIS	Ryt	is	1	0	25	0	2	5	6	8	8	9	9	16	6	19
RIS	Ryt	is	2	0	25	2	4	7	11	12	12	12	12	13	5	20
RIS	Ryt	is	3	0	25	1	4	6	10	11	11	11	11	14	6	19
RIS	Ryt	is	4	0	25	2	6	10	11	12	12	13	13	12	3	22
REN	Ryt	en	1	0	25	5	8	10	14	14	14	14	14	11	5	20
REN	Ryt	en	2	0	25	6	7	13	13	13	13	13	13	12	5	20
REN	Ryt	en	3	0	25	1	4	9	13	13	13	13	13	12	5	20
REN	Ryt	en	4	0	25	2	6	9	11	11	11	11	11	14	9	16
RES	Ryt	es	1	0	25	3	6	9	10	10	10	11	11	14	6	19
RES	Ryt	es	2	0	25	1	6	10	14	15	15	15	15	10	5	20
RES	Ryt	es	3	0	25	2	4	7	12	13	13	13	13	12	6	19
RES	Ryt	es	4	0	25	3	8	12	13	14	14	14	14	11	5	20
RC	Ryt	ctrl	1	-0.6	25	0	0	4	8	12	13	13	13	12	4	21
RC	Ryt	ctrl	2	-0.6	25	0	0	8	10	11	13	13	13	12	5	20
RC	Ryt	ctrl	3	-0.6	25	0	2	5	7	7	7	8	8	17	4	21
RC	Ryt	ctrl	4	-0.6	25	0	2	7	7	7	11	11	11	14	11	14
RIN	Ryt	in	1	-0.6	25	0	3	7	10	10	10	10	10	15	6	19
RIN	Ryt	in	2	-0.6	25	0	1	4	5	8	9	9	9	16	7	18
RIN	Ryt	in	3	-0.6	25	0	2	5	7	9	10	10	10	15	10	15
RIN	Ryt	in	4	-0.6	25	0	2	6	6	9	11	11	11	14	4	21
RIS	Ryt	is	1	-0.6	25	0	2	4	6	7	8	8	8	17	4	21
RIS	Ryt	is	2	-0.6	25	0	2	9	11	13	14	14	14	11	6	19
RIS	Ryt	is	3	-0.6	25	0	5	8	8	8	8	10	10	15	6	19
RIS	Ryt	is	4	-0.6	25	0	3	6	8	10	10	10	10	15	5	20
REN	Ryt	en	1	-0.6	25	0	4	8	10	11	11	12	13	12	6	19
REN	Ryt	en	2	-0.6	25	0	4	9	13	15	16	16	16	9	5	20
REN	Ryt	en	3	-0.6	25	0	4	7	10	12	12	12	12	13	9	16
REN	Ryt	en	4	-0.6	25	0	9	13	18	18	18	18	18	7	3	22
RES	Ryt	es	1	-0.6	25	0	4	8	11	12	12	12	12	13	5	20
RES	Ryt	es	2	-0.6	25	0	4	9	11	16	17	17	17	8	3	22
RES	Ryt	es	3	-0.6	25	0	3	8	9	11	12	13	13	12	8	17
RES	Ryt	es	4	-0.6	25	0	6	12	13	16	16	16	16	9	6	19
RC	Ryt	ctrl	1	-0.9	25	0	0	1	3	4	6	8	9	16	4	21
RC	Ryt	ctrl	2	-0.9	25	0	0	3	4	8	8	11	12	13	5	20
RC	Ryt	ctrl	3	-0.9	25	0	0	2	4	6	6	8	8	17	4	21
RC	Ryt	ctrl	4	-0.9	25	0	0	1	6	8	8	10	10	15	0	25
RIN	Ryt	in	1	-0.9	25	0	0	2	3	3	5	8	8	17	7	18
RIN	Ryt	in	2	-0.9	25	0	0	2	3	8	9	11	11	14	9	16
RIN	Ryt	in	3	-0.9	25	0	0	2	6	9	10	16	16	9	7	18
RIN	Ryt	in	4	-0.9	25	0	0	2	5	5	8	13	13	12	5	20

Code	Sp	Trt	Rep	WP	Tot	2	3	4	5	7	10	15	21	left	dead	totF
RIS	Ryt	is	1	-0.9	25	0	0	2	8	9	11	11	11	14	9	16
RIS	Ryt	is	2	-0.9	25	0	0	2	4	4	5	6	6	19	7	18
RIS	Ryt	is	3	-0.9	25	0	0	2	6	10	11	15	15	10	6	19
RIS	Ryt	is	4	-0.9	25	0	0	2	5	6	9	11	11	14	12	13
REN	Ryt	en	1	-0.9	25	0	0	5	8	10	11	12	12	13	4	21
REN	Ryt	en	2	-0.9	25	0	0	1	5	9	11	11	11	14	9	16
REN	Ryt	en	3	-0.9	25	0	0	2	6	8	12	14	14	11	4	21
REN	Ryt	en	4	-0.9	25	0	0	5	9	11	11	11	11	14	7	18
RES	Ryt	es	1	-0.9	25	0	0	3	4	11	12	12	12	13	6	19
RES	Ryt	es	2	-0.9	25	0	0	4	7	10	13	14	14	11	8	17
RES	Ryt	es	3	-0.9	25	0	0	4	5	10	13	13	13	12	8	17
RES	Ryt	es	4	-0.9	25	0	0	3	7	10	11	15	15	10	6	19
RC	Ryt	ctrl	1	-1.2	25	0	0	0	0	3	3	8	9	16	3	22
RC	Ryt	ctrl	2	-1.2	25	0	0	0	0	2	3	7	8	17	3	22
RC	Ryt	ctrl	3	-1.2	25	0	0	0	0	2	2	3	5	20	10	15
RC	Ryt	ctrl	4	-1.2	25	0	0	0	0	0	1	5	6	19	4	21
RIN	Ryt	in	1	-1.2	25	0	0	0	0	2	3	3	4	21	5	20
RIN	Ryt	in	2	-1.2	25	0	0	0	0	3	5	5	8	17	3	22
RIN	Ryt	in	3	-1.2	25	0	0	0	0	4	5	11	11	14	2	23
RIN	Ryt	in	4	-1.2	25	0	0	0	0	0	0	6	7	18	6	19
RIS	Ryt	is	1	-1.2	25	0	0	0	0	2	3	4	4	21	6	19
RIS	Ryt	is	2	-1.2	25	0	0	0	0	1	1	5	5	20	7	18
RIS	Ryt	is	3	-1.2	25	0	0	0	0	3	5	6	7	18	4	21
RIS	Ryt	is	4	-1.2	25	0	0	0	0	3	4	8	9	16	5	20
REN	Ryt	en	1	-1.2	25	0	0	0	0	2	2	6	7	18	4	21
REN	Ryt	en	2	-1.2	25	0	0	0	0	1	2	3	4	21	5	20
REN	Ryt	en	3	-1.2	25	0	0	0	1	3	5	8	9	16	6	19
REN	Ryt	en	4	-1.2	25	0	0	0	1	5	6	8	8	17	5	20
RES	Ryt	es	1	-1.2	25	0	0	0	0	0	1	5	7	18	5	20
RES	Ryt	es	2	-1.2	25	0	0	0	2	3	5	6	6	19	3	22
RES	Ryt	es	3	-1.2	25	0	0	0	1	1	1	3	4	21	5	20
RES	Ryt	es	4	-1.2	25	0	0	0	0	1	2	6	7	18	2	23

ctrl: untreated control
en: encrusted witouh SA
es: encrusted with SA
in: imbibed witouh SA
is: imbibved with SA

Sumary *Rytidosperma geniculatum* laboratory germiation experiment

WP	Code	Avg D	St.err D	Avg Gt	St.err Gt	Avg G adj	St.err G adj
0	RC	4.00%	2.45%	56.00%	2.00%	58.31%	1.21%
	REN	24.00%	3.46%	51.00%	2.18%	67.19%	1.12%
	RES	22.00%	1.00%	53.00%	2.96%	67.83%	3.11%
	RIN	7.00%	1.66%	46.00%	3.00%	49.45%	3.05%
	RIS	20.00%	2.45%	45.00%	2.96%	56.09%	2.54%
-0.6	RC	24.00%	5.83%	45.00%	4.09%	60.89%	7.29%
	REN	23.00%	4.33%	59.00%	4.77%	76.31%	2.60%
	RES	22.00%	3.61%	58.00%	4.12%	74.49%	4.44%
	RIN	27.00%	4.33%	40.00%	1.41%	55.42%	3.29%
	RIS	21.00%	1.66%	42.00%	4.36%	53.60%	6.41%
-0.9	RC	13.00%	3.84%	39.00%	2.96%	45.24%	4.34%
	REN	24.00%	4.24%	48.00%	2.45%	63.42%	2.29%
	RES	28.00%	2.00%	54.00%	2.24%	75.23%	3.64%
	RIN	28.00%	2.83%	48.00%	5.83%	66.77%	7.88%
	RIS	34.00%	4.58%	43.00%	6.38%	66.41%	9.96%
-1.2	RC	20.00%	5.83%	28.00%	3.16%	34.79%	2.25%
	REN	20.00%	1.41%	28.00%	3.74%	35.18%	5.04%
	RES	15.00%	2.60%	24.00%	2.45%	28.18%	2.73%
	RIN	16.00%	3.16%	30.00%	5.00%	35.26%	4.97%
	RIS	22.00%	2.24%	25.00%	3.84%	31.79%	4.39%

Avg D: average mortality
St.err D: standard error mortality

Avg Gt: average germination (keeping dead seeds in the total)
St.err D: standard error germination (keeping dead seeds in the total)

Avg G adj: average germination adjusted from viability (removing dead seeds from the total)
St.err G adj: standard error germination adjusted from viability (removing dead seeds from the total)
