# Supplementary material for: Seed encrusting with salicylic acid: A novel approach to improve establishment of grass species in ecological restoration
Source: PLoS One. 2021 Jun 9;16(6):e0242035. doi: 10.1371/journal.pone.0242035 (PMC8189473; doi:10.1371/journal.pone.0242035)

AUSTROSTIPA SCABRA

0 MPa

Final germination

	Final	St.Err	Ctrl/trt	No/Sa
Ctrl	0.85	0.017		
No	0.89	0.012	-	
Sa	0.847	0.012	-	*

			Ctrl/trt	Imb/Encr
Ctrl	0.85	0.017		
Imb	0.871	0.012	-	
Encr	0.866	0.012	-	-

			Ctrl/trt	No/Sa	Imb/Encr
Ctrl	0.85	0.017			
IN	0.889	0.017	-		
IS	0.852	0.017	-	-	
EN	0.891	0.016	-		-
ES	0.841	0.016	-	*	-

T50

	Days	St.Err	Ctrl/trt	No/Sa
Ctrl	3.433	0.075		
No	2.956	0.053	-	
Sa	2.842	0.057	-	-

			Ctrl/trt	Imb/Encr
Ctrl	3.433	0.077		
Imb	2.9	0.058	***	
Encr	2.902	0.056	***	-

			Ctrl/trt	No/Sa	Imb/Encr
Ctrl	3.433	0.076			
IN	2.965	0.076	***		
IS	2.824	0.086	***	-	
EN	2.945	0.077	***		-
ES	2.858	0.078	***	*	-

-0.6 MPa

Final germination

	Final	St.Err	Ctrl/trt	No/Sa
Ctrl	0.747	0.02		
No	0.828	0.013	**	
Sa	0.86	0.014	**	-

			Ctrl/trt	Imb/Encr
Ctrl	0.747	0.017		
Imb	0.88	0.011	***	
Encr	0.811	0.012	**	***

			Ctrl/trt	No/Sa	Imb/Encr
Ctrl	0.747	0.015			
IN	0.844	0.014	*		
IS	0.916	0.014	**	-	
EN	0.812	0.015	-		*
ES	0.81	0.016	-	-	*

T50

	Days	St.Err	Ctrl/trt	No/Sa
Ctrl	3.532	0.108		
No	3.395	0.061	-	
Sa	3.382	0.058	-	-

			Ctrl/trt	Imb/Encr
Ctrl	3.533	0.09		
Imb	3.292	0.042	*	
Encr	3.525	0.06		**

			Ctrl/trt	No/Sa	Imb/Encr
Ctrl	3.533	0.083			
IN	3.371	0.06	-		
IS	3.225	0.049	**	-	
EN	3.424	0.074	-		-
ES	3.633	0.082	-	-	***

-0.9 MPa

Final germination

	Final	St.Err	Ctrl/trt	No/Sa
Ctrl	0.709	0.048		
No	0.803	0.025	-	
Sa	0.865	0.026	**	-

			Ctrl/trt	Imb/Encr
Ctrl	0.709	0.047		
Imb	0.852	0.025	**	
Encr	0.817	0.026	*	-

			Ctrl/trt	No/Sa	Imb/Encr
Ctrl	0.709	0.047			
IN	0.794	0.034	-		
IS	0.908	0.035	***	*	
EN	0.81	0.035	-		-
ES	0.823	0.038	*	-	-

T50

	Days	St.Err	Ctrl/trt	No/Sa
Ctrl	5.396	0.427		
No	3.99	0.138	**	
Sa	4.216	0.143	*	-

			Ctrl/trt	Imb/Encr
Ctrl	5.396	0.421		
Imb	3.957	0.126	**	
Encr	4.274	0.155	*	-

			Ctrl/trt	No/Sa	Imb/Encr
Ctrl	5.396	0.416			
IN	3.938	0.183	**		
IS	3.972	0.169	**	-	
EN	4.042	0.197	**		-
ES	4.519	0.233	-	-	*

- not significant, * P<0.05, ** P<0.01, *** P<0.001
Red worst than ctlr, green better than ctrl
Ctrl:control - IN:ImbibedNoSA - IS:ImbibedSA - EN:EncrustedNoSa - ES:EncrustedSa
No:IN+EN - SA:IS+ES - Imb:IN+IS - Encr:EN+ES

-1.2 MPa

Final germination

	Final	St.Err	Ctrl/trt	No/Sa
Ctrl	0.515	0.049		
No	0.565	0.02	-	
Sa	0.578	0.018	-	-

	Final	St.Err	Ctrl/trt	Imb/Encr
Ctrl	0.515	0.049		
Imb	0.554	0.016	-	
Encr	0.596	0.022	-	-

	Final	St.Err	Ctrl/trt	No/Sa	Imb/Encr
Ctrl	0.515	0.047			
IN	0.514	0.024	-		
IS	0.596	0.021	-	**	
EN	0.617	0.03	-		**
ES	0.573	0.03	-	-	-

T50

	Days	St.Err	Ctrl/trt	No/Sa
Ctrl	8.503	0.893		
No	6.88	0.261	-	
Sa	6.227	0.211	*	-

	Days	St.Err	Ctrl/trt	Imb/Encr
Ctrl	8.496	0.892		
Imb	6.203	0.179	*	
Encr	7.006	0.295	-	*

	Days	St.Err	Ctrl/trt	No/Sa	Imb/Encr
Ctrl	8.503	0.85			
IN	6.551	0.293	*		
IS	5.929	0.209	**	-	
EN	7.208	0.38	-		-
ES	6.772	0.409	-	-	*

Emrg

emergence

	Final	St.Err	Ctrl/trt	No/Sa
Ctrl	0.523	0.015		
No	0.51	0.011	-	
Sa	0.444	0.011	***	***

	Final	St.Err	Ctrl/trt	Imb/Encr
Ctrl	0.523	0.016		
Imb	0.502	0.011		
Encr	0.452	0.011	***	**

	Final	St.Err	Ctrl/trt	No/Sa	Imb/Encr
Ctrl	0.523	0.015			
IN	0.532	0.015	-		
IS	0.472	0.015	*	**	
EN	0.489	0.015	-		*
ES	0.416	0.016	***	**	**

T50

	Weeks	St.Err	Ctrl/trt	No/Sa
Ctrl	1.635	0.332		
No	1.633	0.199	-	
Sa	1.641	0.151	-	-

	Weeks	St.Err	Ctrl/trt	Imb/Encr
Ctrl	1.635	0.343		
Imb	1.652	0.194	-	
Encr	1.623	0.172	-	-

	Weeks	St.Err	Ctrl/trt	No/Sa	Imb/Encr
Ctrl	1.636	0.332			
IN	1.648	0.213	-		
IS	1.641	0.245	-	-	
EN	1.599	0.265	-		-
ES	1.651	0.198	-	-	-

- not significant, * $P<0.05$, ** $P<0.01$, *** $P<0.001$
Red worst than ctlr, green better than ctrl
Ctrl:control - IN:ImbibedNoSA - IS:ImbibedSA - EN:EncrustedNoSa - ES:EncrustedSa
No:IN+EN - SA:IS+ES - Imb:IN+IS - Encr:EN+ES

Austrostipa scabra

0.0 MPa

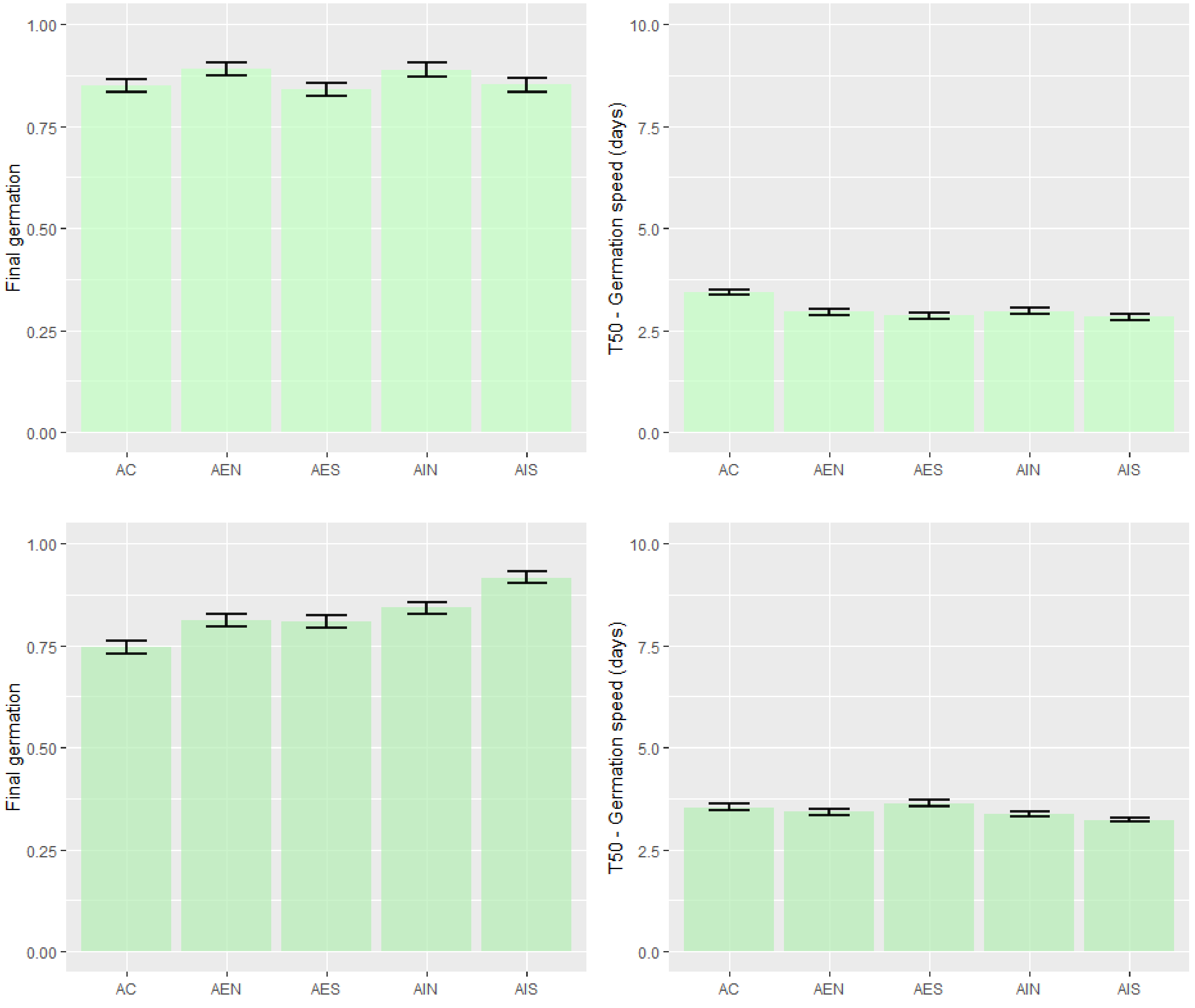

-0.9 MPa

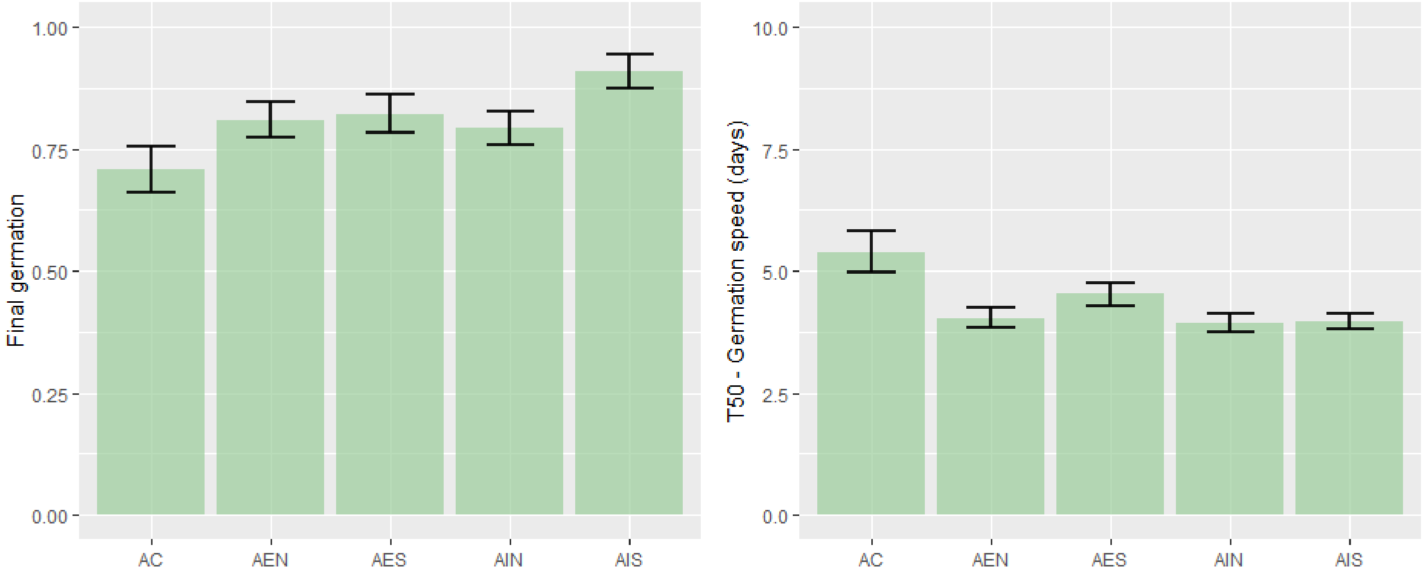

Austrostipa scabra

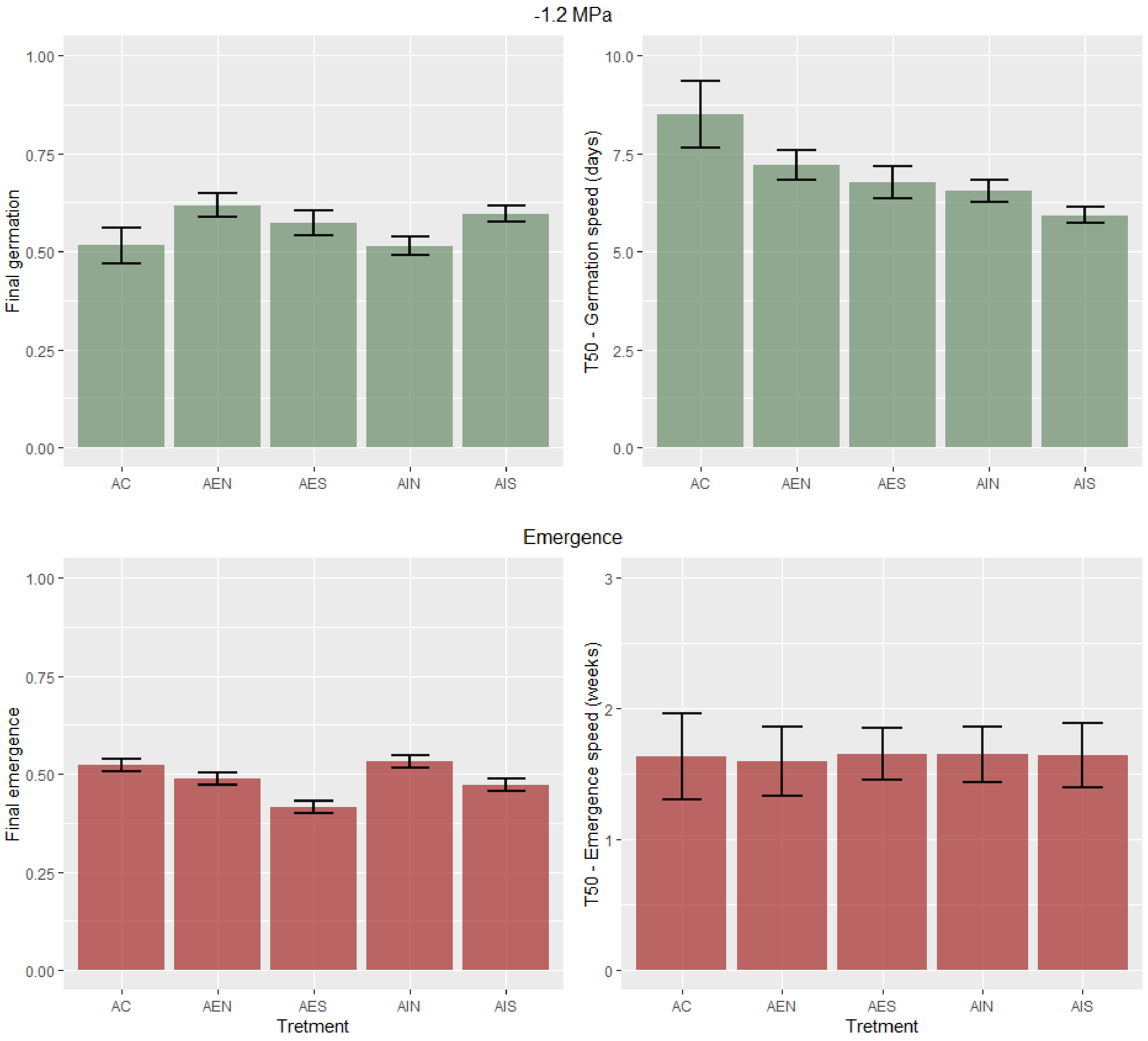

MICROLAENA STIPOIDES

0 MPa

Final germination

	Final	St.Err	No/Sa	
Ctrl	0.73	0.03		
No	0.749	0.022	-	
Sa	0.67	0.023	-	*

			Ctrl/trt	Imb/Encr
Ctrl	0.73	0.021		
Imb	0.605	0.016	***	
Encr	0.814	0.015	**	***

			Ctrl/trt	No/Sa	Imb/Encr
Ctrl	0.73	0.02			
IN	0.641	0.022	**		
IS	0.569	0.022	***	*	
EN	0.858	0.019	***		***
ES	0.77	0.021	-	**	***

T50

	Days	St.Err	No/Sa	
Ctrl	2.893	0.17		
No	2.713	0.117	-	
Sa	2.715	0.141	-	-

			Ctrl/trt	Imb/Encr
Ctrl	2.893	0.12		
Imb	2.943	0.113	-	
Encr	2.556	0.074	*	**

			Ctrl/trt	No/Sa	Imb/Encr
Ctrl	2.893	0.112			
IN	2.947	0.139	-		
IS	2.94	0.161	-	-	
EN	2.558	0.088	*		*
ES	2.553	0.111	*	-	-

-0.6 MPa

Final germination

	Final	St.Err	No/Sa	
Ctrl	0.743	0.028		
No	0.774	0.019	-	
Sa	0.861	0.019	***	***

			Ctrl/trt	Imb/Encr
Ctrl	0.743	0.026		
Imb	0.78	0.018	-	
Encr	0.854	0.017	***	**

			Ctrl/trt	No/Sa	Imb/Encr
Ctrl	0.743	0.024			
IN	0.762	0.025	-		
IS	0.795	0.023	-	-	
EN	0.787	0.022	-		-
ES	0.922	0.023	***	***	***

T50

	Days	St.Err	No/Sa	
Ctrl	3.374	0.159		
No	3.249	0.097	-	
Sa	3.385	0.092	-	-

			Ctrl/trt	Imb/Encr
Ctrl	3.374	0.149		
Imb	3.519	0.097	-	
Encr	3.144	0.078	-	**

			Ctrl/trt	No/Sa	Imb/Encr
Ctrl	3.374	0.139			
IN	3.371	0.132	-		
IS	3.658	0.123	-	-	
EN	3.143	0.109	-		-
ES	3.146	0.098	-	-	**

-0.9 MPa

Final germination

	Final	St.Err	No/Sa	
Ctrl	0.745	0.023		
No	0.792	0.017	-	
Sa	0.736	0.016	-	*

			Ctrl/trt	Imb/Encr
Ctrl	0.745	0.02		
Imb	0.703	0.014	-	
Encr	0.823	0.014	**	***

			Ctrl/trt	No/Sa	Imb/Encr
Ctrl	0.745	0.019			
IN	0.73	0.021	-		
IS	0.684	0.018	*	-	
EN	0.856	0.019	***		***
ES	0.789	0.019	-	*	***

T50

	Days	St.Err	No/Sa	
Ctrl	4.357	0.117		
No	4.305	0.087	-	
Sa	4.14	0.073	-	-

			Ctrl/trt	Imb/Encr
Ctrl	4.358	0.101		
Imb	4.311	0.075	-	
Encr	4.13	0.063	-	-

			Ctrl/trt	No/Sa	Imb/Encr
Ctrl	4.358	0.096			
IN	4.607	0.137	-		
IS	4.11	0.08	-	**	
EN	4.085	0.08	**		**
ES	4.177	0.088	-	-	-

- not significant, * P<0.05, ** P<0.01, *** P<0.001
Red worst than ctlr, green better than ctrl
Ctrl:control - IN:ImbibedNoSA - IS:ImbibedSA - EN:EncrustedNoSa - ES:EncrustedSa
No:IN+EN - SA:IS+ES - Imb:IN+IS - Encr:EN+ES

-1.2 MPa

Final germination

	Final	St.Err	No/Sa	
Ctrl	0.675	0.042		
No	0.676	0.025	-	
Sa	0.564	0.023	*	**

			Ctrl/trt	Imb/Encr
Ctrl	0.675	0.037		
Imb	0.54	0.022	**	
Encr	0.702	0.02	-	***

			Ctrl/trt	No/Sa	Imb/Encr
Ctrl	0.675	0.035			
IN	0.621	0.037	***		
IS	0.47	0.025	-	**	
EN	0.744	0.026	-		**
ES	0.66	0.028	-	*	***

T50

	Days	St.Err	No/Sa	
Ctrl	7.094	0.503		
No	6.515	0.272	-	
Sa	5.973	0.262	-	-

			Ctrl/trt	Imb/Encr
Ctrl	7.093	0.439		
Imb	6.423	0.303	-	
Encr	6.151	0.194	-	-

			Ctrl/trt	No/Sa	Imb/Encr
Ctrl	7.093	0.421			
IN	7.103	0.482	-		
IS	5.842	0.33	*	*	
EN	6.208	0.239	-		-
ES	6.09	0.293	-	-	-

Emrg

emergence

	Final	St.Err	Ctrl/trt	No/Sa
Ctrl	0.35	0.019		
No	0.443	0.013	***	
Sa	0.426	0.012	***	-

			Ctrl/trt	Imb/Encr
Ctrl	0.35	0.016		
Imb	0.39	0.011	*	
Encr	0.479	0.01	***	***

			Ctrl/trt	No/Sa	Imb/Encr
Ctrl	0.349	0.016			
IN	0.407	0.017	**		
IS	0.376	0.014	-	-	
EN	0.484	0.014	***		**
ES	0.476	0.015	***	-	***

T50

	Weeks	St.Err	Ctrl/trt	No/Sa
Ctrl	1.739	0.162		
No	1.733	0.098	-	
Sa	1.713	0.112	-	-

			Ctrl/trt	Imb/Encr
Ctrl	1.739	0.138		
Imb	1.773	0.09	-	
Encr	1.688	0.092	-	-

			Ctrl/trt	No/Sa	Imb/Encr
Ctrl	1.74	0.14			
IN	1.862	0.123	-		
IS	1.727	0.21	-	-	
EN	1.68	0.2	-		-
ES	1.712	0.105	-	-	-

- not significant, * P<0.05, ** P<0.01, *** P<0.001
Red worst than ctlr, green better than ctrl
Ctrl:control - IN:ImbibedNoSA - IS:ImbibedSA - EN:EncrustedNoSa - ES:EncrustedSa
No:IN+EN - SA:IS+ES - Imb:IN+IS - Encr:EN+ES

Microlaena stipoides

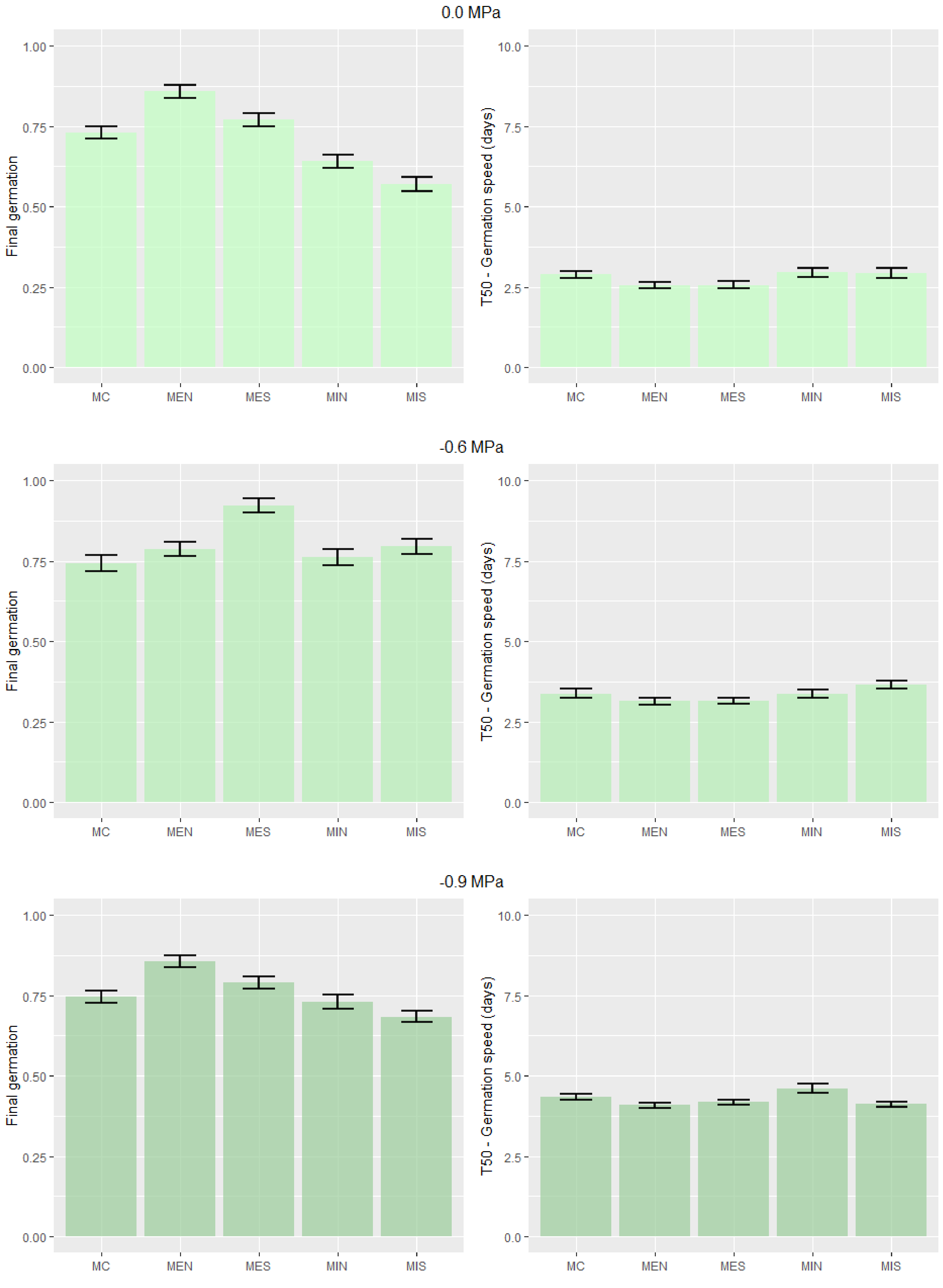

Microlaena stipoides

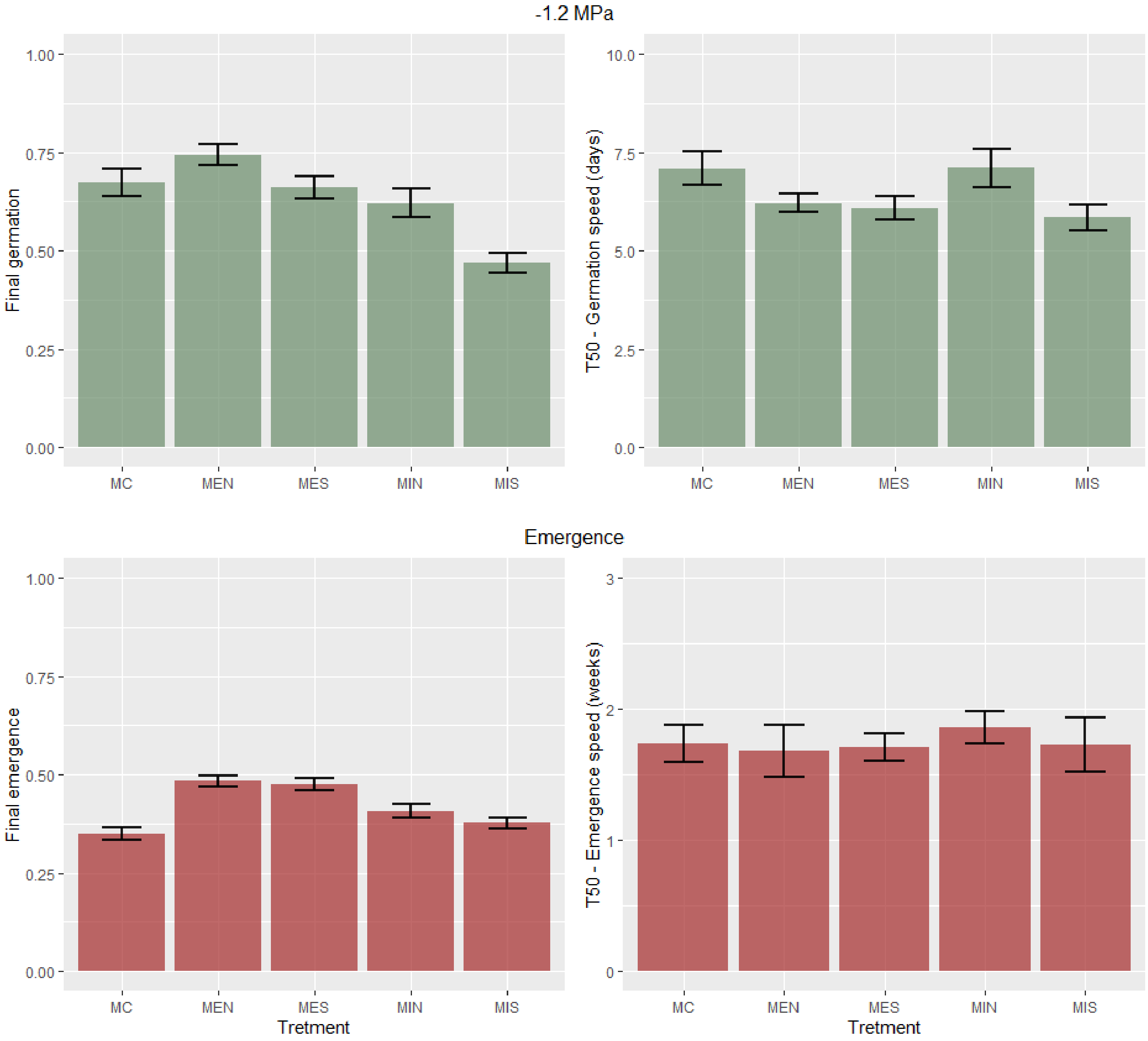

RYTIDOSPERMA GENICULATUM

0 MPa

Final germination

	Final	St.Err	No/Sa	
Ctrl	0.583	0.023		
No	0.592	0.015	-	
Sa	0.612	0.017	-	-

			Ctrl/trt	Imb/Encr
Ctrl	0.583	0.017		
Imb	0.522	0.012	**	
Encr	0.681	0.012	***	**

			Ctrl/trt	No/Sa	Imb/Encr
Ctrl	0.583	0.017			
IN	0.506	0.015	**		
IS	0.54	0.017	-	-	
EN	0.678	0.016	***		***
ES	0.684	0.016	***	-	***

T50

	Days	St.Err	No/Sa	
Ctrl	3.178	0.181		
No	2.887	0.118	-	
Sa	3.283	0.121	-	*

			Ctrl/trt	Imb/Encr
Ctrl	3.178	0.132		
Imb	3.196	0.099		
Encr	2.987	0.078		**

			Ctrl/trt	No/Sa	Imb/Encr
Ctrl	3.178	0.13			
IN	2.968	0.129	-		
IS	3.45	0.143	-	**	
EN	2.81	0.11	*		-
ES	3.157	0.107	-	*	-

-0.6 MPa

Final germination

	Final	St.Err	No/Sa	
Ctrl	0.589	0.039		
No	0.643	0.024	-	
Sa	0.633	0.025	-	-

			Ctrl/trt	Imb/Encr
Ctrl	0.589	0.03		
Imb	0.531	0.019	-	
Encr	0.745	0.018	***	***

			Ctrl/trt	No/Sa	Imb/Encr
Ctrl	0.589	0.03			
IN	0.534	0.029	-		
IS	0.528	0.027	-	-	
EN	0.754	0.025	***		***
ES	0.737	0.027	***	-	***

T50

	Days	St.Err	No/Sa	
Ctrl	4.023	0.299		
No	3.662	0.168	-	
Sa	3.628	0.173	-	-

			Ctrl/trt	Imb/Encr
Ctrl	4.026	0.229		
Imb	3.775	0.166	-	
Encr	3.559	0.108	-	-

			Ctrl/trt	No/Sa	Imb/Encr
Ctrl	4.023	0.23			
IN	3.981	0.257	-		
IS	3.588	0.215	-	-	
EN	3.475	0.144	-		-
ES	3.658	0.166	-	-	-

-0.9 MPa

Final germination

	Final	St.Err	No/Sa	
Ctrl	0.446	0.054		
No	0.641	0.03	***	
Sa	0.691	0.026	***	-

			Ctrl/trt	Imb/Encr
Ctrl	0.447	0.053		
Imb	0.67	0.034	***	
Encr	0.673	0.024	***	-

			Ctrl/trt	No/Sa	Imb/Encr
Ctrl	0.447	0.052			
IN	0.704	0.061	***		
IS	0.651	0.037	***	-	
EN	0.613	0.031	**		-
ES	0.732	0.033	***	**	-

T50

	Days	St.Err	No/Sa	
Ctrl	5.958	0.932		
No	5.596	0.333	-	
Sa	5.236	0.229	-	-

			Ctrl/trt	Imb/Encr
Ctrl	5.961	0.926		
Imb	5.981	0.38	-	
Encr	5.027	0.202	-	*

			Ctrl/trt	No/Sa	Imb/Encr
Ctrl	5.962	0.9			
IN	6.992	0.76	-		
IS	5.265	0.366	-	-	
EN	4.802	0.267	-		*
ES	5.219	0.275	-	-	-

- not significant, * P<0.05, ** P<0.01, *** P<0.001
Red worst than ctlr, green better than ctrl
Ctrl:control - IN:ImbibedNoSA - IS:ImbibedSA - EN:EncrustedNoSa - ES:EncrustedSa
No:IN+EN - SA:IS+ES - Imb:IN+IS - Encr:EN+ES

-1.2 MPa

Final germination

	Final	St.Err	No/Sa	
Ctrl	0.404	0.089		
No	0.376	0.035	-	
Sa	0.329	0.037	-	-

			Ctrl/trt	Imb/Encr
Ctrl	0.404	0.09		
Imb	0.361	0.033	-	
Encr	0.347	0.042	-	-

			Ctrl/trt	No/Sa	Imb/Encr
Ctrl	0.404	0.088			
IN	0.385	0.056	-		
IS	0.341	0.038	-	-	
EN	0.368	0.043	-		-
ES	0.328	0.083	-	-	-

T50

	Days	St.Err	No/Sa	
Ctrl	12.59	2.391		
No	10.03	0.923	-	
Sa	10.49	1.118	-	-

			Ctrl/trt	Imb/Encr
Ctrl	12.59	2.422		
Imb	10.06	0.879	-	
Encr	10.51	1.249	-	-

			Ctrl/trt	No/Sa	Imb/Encr
Ctrl	12.59	2.367			
IN	10.71	1.449	-		
IS	9.494	1.034	-	-	
EN	9.362	1.128	-		-
ES	12.07	2.758	-	-	-

Emrg

emergence

	Final	St.Err	Ctrl/trt	No/Sa
Ctrl	0.283	0.024		
No	0.297	0.013	-	
Sa	0.312	0.015	-	-

			Ctrl/trt	Imb/Encr
Ctrl	0.283	0.024		
Imb	0.307	0.015	-	
Encr	0.303	0.012	-	-

			Ctrl/trt	No/Sa	Imb/Encr
Ctrl	0.283	0.024			
IN	0.312	0.019	-		
IS	0.304	0.026	-	-	
EN	0.283	0.017	-		-
ES	0.323	0.018	-	-	-

T50

	Weeks	St.Err	Ctrl/trt	No/Sa
Ctrl	2.265	0.313		
No	1.889	0.133	-	
Sa	2.04	0.156	-	-

			Ctrl/trt	Imb/Encr
Ctrl	2.265	0.312		
Imb	2.068	0.165	-	
Encr	1.874	0.124	-	-

			Ctrl/trt	No/Sa	Imb/Encr
Ctrl	2.265	0.31			
IN	1.948	0.194	-		
IS	2.228	0.293	-	-	
EN	1.832	0.176	-		-
ES	1.913	0.172	-	-	-

- not significant, * P<0.05, ** P<0.01, *** P<0.001
Red worst than ctlr, green better than ctrl
Ctrl:control - IN:ImbibedNoSA - IS:ImbibedSA - EN:EncrustedNoSa - ES:EncrustedSa
No:IN+EN - SA:IS+ES - Imb:IN+IS - Encr:EN+ES

Rytidosperma geniculatum

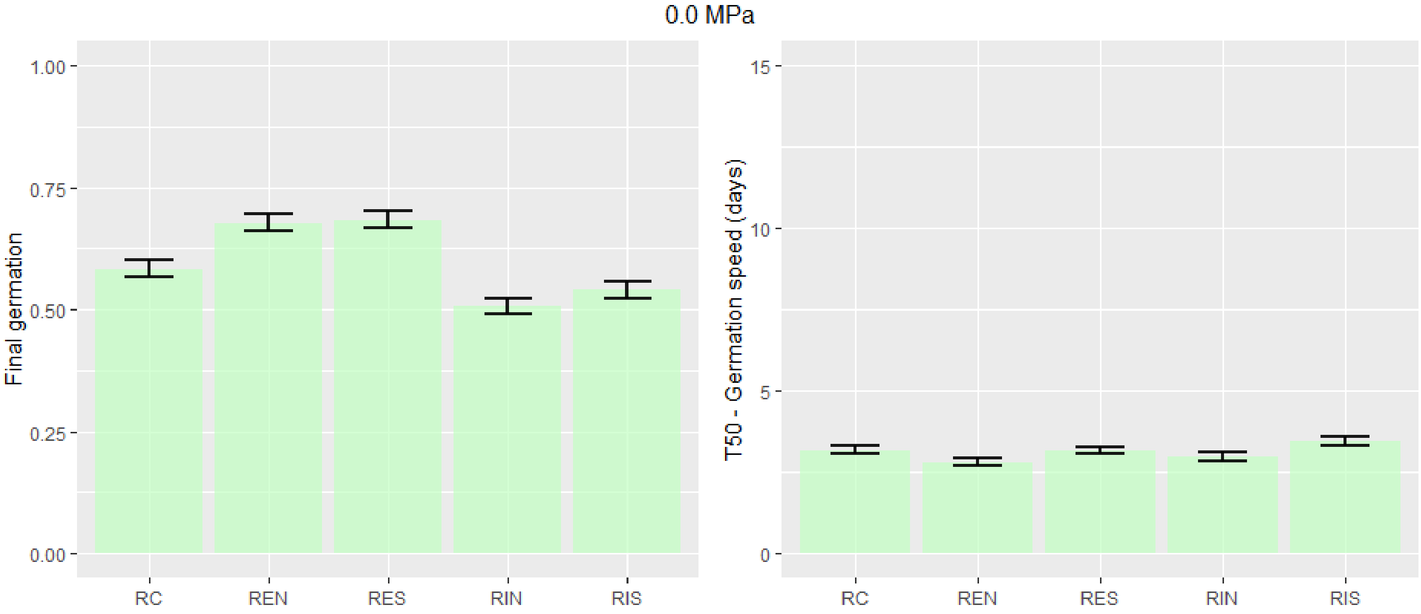

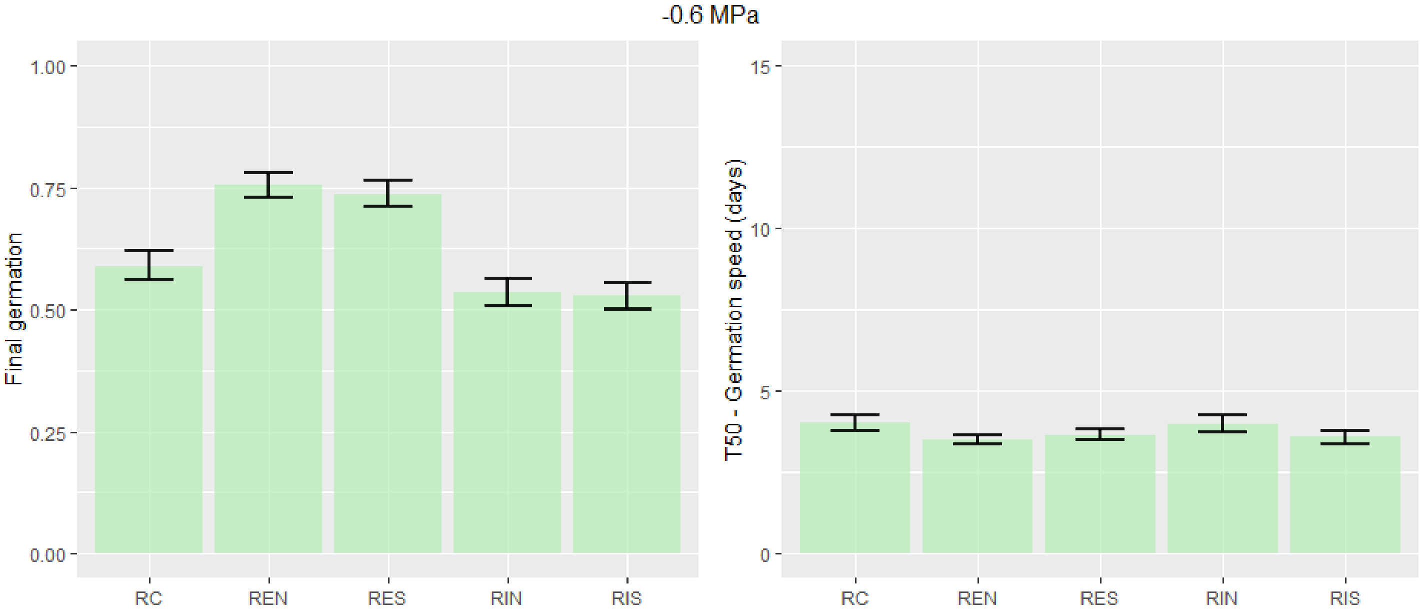

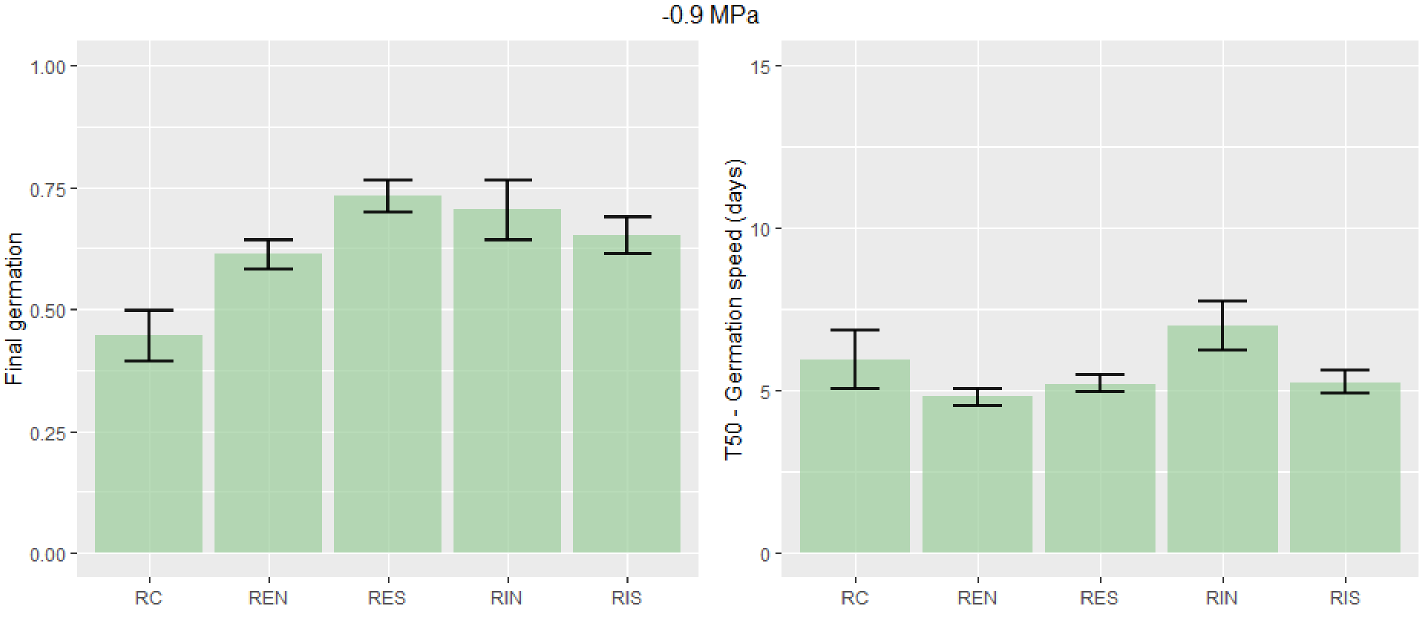

Rytidosperma geniculatum

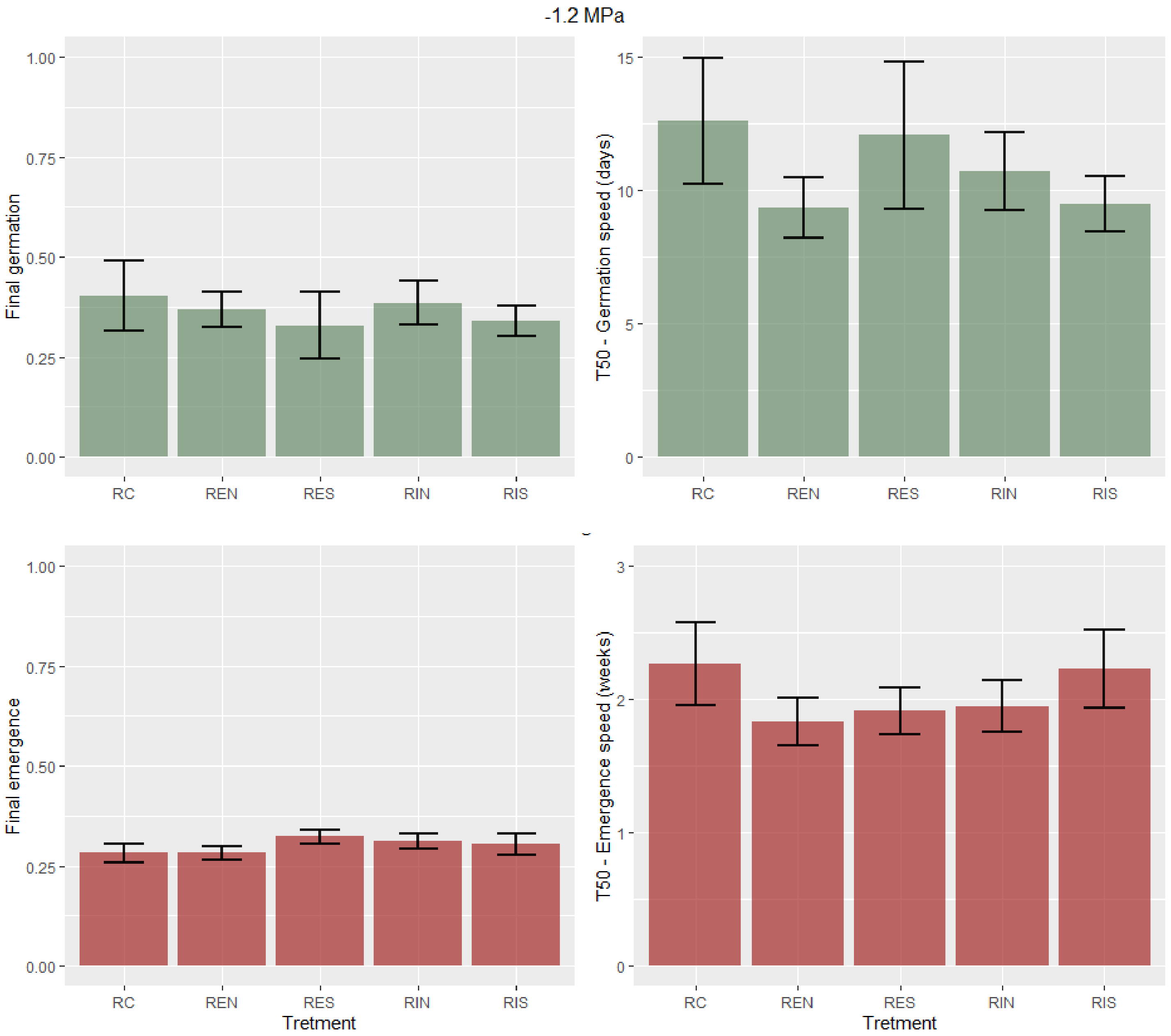

Supplement: S4 File — Statistics obtained with parameter comparison of DRM model comparing treatment, SA, and combination of treatment and SA against the untreated control. (PDF) [file pone.0242035.s004.pdf]
