# Supplementary material for: Seed encrusting with salicylic acid: A novel approach to improve establishment of grass species in ecological restoration
Source: PLoS One. 2021 Jun 9;16(6):e0242035. doi: 10.1371/journal.pone.0242035 (PMC8189473; doi:10.1371/journal.pone.0242035)
Supplement: S5 File — Summary table are provided for each experiment with average and standard errors for germination (germination bag experiment), emergence (line experiment), and survival (line and plot experiments). (PDF) [file pone.0242035.s005.pdf]

GERMINATION BAGS EXPERIMENT						
Rep	Code	Species	Trt	grm	no	tot
1	AC	Aus	ctrl	22	14	36
1	AEN	Aus	en	26	23	49
1	AES	Aus	es	32	11	43
1	AIN	Aus	in	32	17	49
1	AIS	Aus	is	35	17	52
1	MC	Mic	ctrl	30	22	52
1	MEN	Mic	en	34	19	53
1	MES	Mic	es	28	23	51
1	MIN	Mic	in	27	23	50
1	MIS	Mic	is	25	25	50
1	RC	Ryt	ctrl	11	27	38
1	REN	Ryt	en	20	29	49
1	RES	Ryt	es	19	27	46
1	RIN	Ryt	in	19	25	44
1	RIS	Ryt	is	14	30	44
2	AC	Aus	ctrl	31	16	47
2	AEN	Aus	en	25	13	38
2	AES	Aus	es	24	15	39
2	AIN	Aus	in	33	9	42
2	AIS	Aus	is	30	22	52
2	MC	Mic	ctrl	34	15	49
2	MEN	Mic	en	29	24	53
2	MES	Mic	es	29	17	46
2	MIN	Mic	in	25	25	50
2	MIS	Mic	is	19	28	47
2	RC	Ryt	ctrl	19	26	45
2	REN	Ryt	en	19	32	51
2	RES	Ryt	es	14	29	43
2	RIN	Ryt	in	21	26	47
2	RIS	Ryt	is	21	28	49
3	AC	Aus	ctrl	30	13	43
3	AEN	Aus	en	26	9	35
3	AES	Aus	es	32	13	45
3	AIN	Aus	in	31	15	46
3	AIS	Aus	is	30	9	39
3	MC	Mic	ctrl	25	25	50
3	MEN	Mic	en	25	25	50
3	MES	Mic	es	33	18	51
3	MIN	Mic	in	27	24	51
3	MIS	Mic	is	26	25	51
3	RC	Ryt	ctrl	23	21	44
3	REN	Ryt	en	18	29	47
3	RES	Ryt	es	16	26	42
3	RIN	Ryt	in	20	28	48
3	RIS	Ryt	is	20	26	46

Buried	9/05/2017
Retrieved	29/05/2017

Aus: Austrostipa scabra
Mic: Microlaena stipoides
Ryt: Rytidosperma geniculatum

ctrl: untreated control
en: encrusted witouh SA
es: encrusted with SA
in: imbibed witouh SA
is: imbibved with SA

GERMINATION BAGS EXPERIMENT						
Rep	Code	Species	Trt	grm	no	tot
4	AC	Aus	ctrl	27	13	40
4	AEN	Aus	en	27	15	42
4	AES	Aus	es	35	12	47
4	AIN	Aus	in	35	11	46
4	AIS	Aus	is	32	10	42
4	MC	Mic	ctrl	26	27	53
4	MEN	Mic	en	26	21	47
4	MES	Mic	es	30	18	48
4	MIN	Mic	in	19	26	45
4	MIS	Mic	is	25	18	43
4	RC	Ryt	ctrl	20	14	34
4	REN	Ryt	en	17	31	48
4	RES	Ryt	es	18	25	43
4	RIN	Ryt	in	15	35	50
4	RIS	Ryt	is	26	22	48

Buried	9/05/2017
Retrieved	29/05/2017

Aus: Austrostipa scabra
Mic: Microlaena stipoides
Ryt: Rytidosperma geniculatum

ctrl: untreated control
en: encrusted witouh SA
es: encrusted with SA
in: imbibed witouh SA
is: imbibved with SA

GERMIANTION
4 WEEKS

Code	Species	Trt	Avg	St.err
AC	Aus	ctrl	66.08%	1.59%
AEN	Aus	en	64.36%	3.78%
AES	Aus	es	70.38%	2.64%
AIN	Aus	in	71.84%	2.80%
AIS	Aus	is	69.53%	3.91%
MC	Mic	ctrl	56.53%	4.07%
MEN	Mic	en	56.05%	2.56%
MES	Mic	es	61.29%	1.89%
MIN	Mic	in	49.79%	2.30%
MIS	Mic	is	49.89%	3.15%
RC	Ryt	ctrl	45.57%	5.64%
REN	Ryt	en	37.95%	0.98%
RES	Ryt	es	38.45%	1.85%
RIN	Ryt	in	39.88%	2.90%
RIS	Ryt	is	43.08%	3.95%

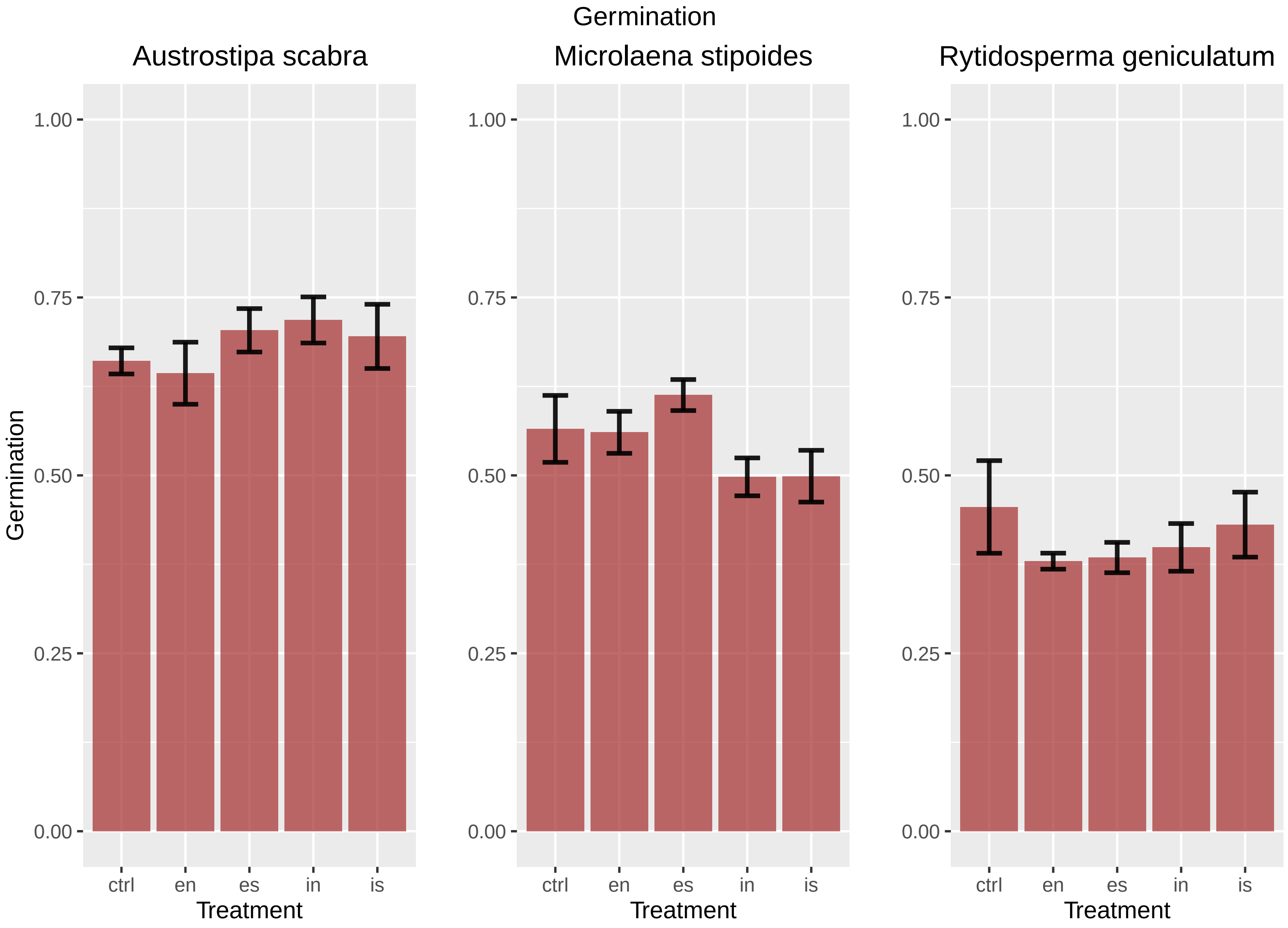
Germination
Austrostipa scabra
Microlaena stipoides
Rytidosperma geniculatum
Germination
1.00
0.75
0.50
0.25
0.00
ctrl
en
es
in
is
Treatment

LINE EXPERIMENT											
Rep	Code	Species	Trt	w1	w2	w3	w4	w6	w8	w10	w45
1	AC	Aus	ctrl	0	40	49	51	51	51	53	19
1	AEN	Aus	en	0	44	44	50	50	50	50	26
1	AES	Aus	es	0	42	46	46	46	47	47	21
1	AIN	Aus	in	0	47	56	56	60	60	60	13
1	AIS	Aus	is	0	52	52	53	59	59	59	19
1	MC	Mic	ctrl	0	24	26	29	30	30	30	11
1	MEN	Mic	en	0	27	37	38	40	40	40	13
1	MES	Mic	es	0	29	29	46	47	47	47	26
1	MIN	Mic	in	0	24	35	40	42	46	46	10
1	MIS	Mic	is	0	34	44	44	45	45	47	23
1	RC	Ryt	ctrl	0	15	19	32	36	36	37	12
1	REN	Ryt	en	0	23	26	27	33	33	36	15
1	RES	Ryt	es	0	19	31	32	40	40	45	14
1	RIN	Ryt	in	0	23	24	33	33	40	40	10
1	RIS	Ryt	is	0	21	31	36	36	48	48	7
2	AC	Aus	ctrl	0	58	58	58	58	60	63	15
2	AEN	Aus	en	0	41	46	46	49	49	49	8
2	AES	Aus	es	0	19	20	21	34	34	34	15
2	AIN	Aus	in	0	45	45	45	45	45	51	14
2	AIS	Aus	is	0	46	47	47	47	47	47	15
2	MC	Mic	ctrl	0	36	45	47	47	47	53	15
2	MEN	Mic	en	0	54	54	54	54	54	54	14
2	MES	Mic	es	0	37	50	53	53	53	53	19
2	MIN	Mic	in	0	18	33	33	33	36	37	15
2	MIS	Mic	is	0	32	40	40	40	40	40	16
2	RC	Ryt	ctrl	0	17	20	20	23	25	25	12
2	REN	Ryt	en	0	16	24	24	29	30	30	10
2	RES	Ryt	es	0	23	28	28	30	30	37	16
2	RIN	Ryt	in	0	15	27	28	31	35	37	11
2	RIS	Ryt	is	0	10	20	20	22	40	40	13
3	AC	Aus	ctrl	0	52	55	59	60	60	60	26
3	AEN	Aus	en	0	52	52	53	60	60	60	11
3	AES	Aus	es	0	43	45	45	54	54	54	27
3	AIN	Aus	in	0	41	48	48	50	51	51	21
3	AIS	Aus	is	0	32	41	41	47	47	47	20
3	MC	Mic	ctrl	0	23	25	27	28	31	31	17
3	MEN	Mic	en	0	49	52	52	53	53	55	18
3	MES	Mic	es	0	37	48	48	51	51	52	24
3	MIN	Mic	in	0	32	39	47	47	48	49	13
3	MIS	Mic	is	0	31	38	38	40	40	43	22
3	RC	Ryt	ctrl	0	16	17	20	25	30	30	8
3	REN	Ryt	en	0	21	21	21	27	27	27	8
3	RES	Ryt	es	0	19	26	28	31	32	32	22
3	RIN	Ryt	in	0	21	25	25	30	32	32	13
3	RIS	Ryt	is	0	14	17	20	21	23	23	18

Start	9/05/2017
w1	15/05/2017
w2	22/05/2017
w3	29/05/2017
w4	4/06/2017
w6	19/06/2017
w8	2/07/2017
w10	16/07/2017
w45- final	11/03/2018

Aus: Austrostipa scabra
Mic: Microlaena stipoides
Ryt: Rytidosperma geniculatum

ctrl: untreated control
en: encrusted witouh SA
es: encrusted with SA
in: imbibed witouh SA
is: imbibved with SA

LINE EXPERIMENT											
Rep	Code	Species	Trt	w1	w2	w3	w4	w6	w8	w10	w45
4	AC	Aus	ctrl	0	34	38	39	39	39	44	11
4	AEN	Aus	en	0	38	40	40	40	44	44	15
4	AES	Aus	es	0	32	40	40	40	40	41	22
4	AIN	Aus	in	0	47	56	56	56	62	62	21
4	AIS	Aus	is	0	31	39	40	40	42	42	21
4	MC	Mic	ctrl	0	16	29	31	34	35	39	15
4	MEN	Mic	en	0	27	42	44	49	49	52	21
4	MES	Mic	es	0	37	42	42	43	43	43	20
4	MIN	Mic	in	0	25	35	35	36	38	38	16
4	MIS	Mic	is	0	17	24	24	26	26	26	11
4	RC	Ryt	ctrl	0	11	15	17	18	21	21	15
4	REN	Ryt	en	0	13	24	24	24	27	27	10
4	RES	Ryt	es	0	17	20	22	22	27	28	14
4	RIN	Ryt	in	0	16	20	20	23	24	24	8
4	RIS	Ryt	is	0	16	18	18	20	24	24	15

Start	9/05/2017
w1	15/05/2017
w2	22/05/2017
w3	29/05/2017
w4	4/06/2017
w6	19/06/2017
w8	2/07/2017
w10	16/07/2017
w45- final	11/03/2018

Aus: Austrostipa scabra
Mic: Microlaena stipoides
Ryt: Rytidosperma geniculatum

ctrl: untreated control
en: encrusted witouh SA
es: encrusted with SA
in: imbibed witouh SA
is: imbibved with SA

			EMERGENCE 10 WEEKS		SURVIVAL 45 WEEKS	
Code	Species	Trt	Avg	St.err	Avg	St.err
AC	Aus	ctrl	55.00%	3.66%	17.75%	2.77%
AEN	Aus	en	50.75%	2.90%	15.00%	3.41%
AES	Aus	es	44.00%	3.69%	21.25%	2.13%
AIN	Aus	in	56.00%	2.52%	17.25%	1.88%
AIS	Aus	is	48.75%	3.13%	18.75%	1.14%
MC	Mic	ctrl	38.25%	4.60%	14.50%	1.09%
MEN	Mic	en	50.25%	3.01%	16.50%	1.60%
MES	Mic	es	48.75%	2.01%	22.25%	1.43%
MIN	Mic	in	42.50%	2.56%	13.50%	1.15%
MIS	Mic	is	39.00%	3.95%	18.00%	2.42%
RC	Ryt	ctrl	28.25%	2.99%	11.75%	1.24%
REN	Ryt	en	30.00%	1.84%	10.75%	1.29%
RES	Ryt	es	35.50%	3.17%	16.50%	1.64%
RIN	Ryt	in	33.25%	3.03%	10.50%	0.90%
RIS	Ryt	is	33.75%	5.32%	13.25%	2.01%

PLOT EXPERIMENT					
Rep	Code	Species	Trt	Initial	Survived w45
1	AC	Aus	ctrl	10	9
1	AEN	Aus	en	10	10
1	AES	Aus	es	10	10
1	AIN	Aus	in	10	8
1	AIS	Aus	is	10	10
1	MC	Mic	ctrl	10	9
1	MEN	Mic	en	10	10
1	MES	Mic	es	10	10
1	MIN	Mic	in	10	6
1	MIS	Mic	is	10	10
1	RC	Ryt	ctrl	10	8
1	REN	Ryt	en	10	6
1	RES	Ryt	es	10	10
1	RIN	Ryt	in	10	6
1	RIS	Ryt	is	10	9
2	AC	Aus	ctrl	10	10
2	AEN	Aus	en	10	10
2	AES	Aus	es	10	10
2	AIN	Aus	in	10	10
2	AIS	Aus	is	10	10
2	MC	Mic	ctrl	10	7
2	MEN	Mic	en	10	10
2	MES	Mic	es	10	10
2	MIN	Mic	in	10	10
2	MIS	Mic	is	10	10
2	RC	Ryt	ctrl	10	9
2	REN	Ryt	en	10	10
2	RES	Ryt	es	10	10
2	RIN	Ryt	in	10	10
2	RIS	Ryt	is	10	9
3	AC	Aus	ctrl	10	10
3	AEN	Aus	en	10	9
3	AES	Aus	es	10	9
3	AIN	Aus	in	10	7
3	AIS	Aus	is	10	9
3	MC	Mic	ctrl	10	10
3	MEN	Mic	en	10	10
3	MES	Mic	es	10	10
3	MIN	Mic	in	10	10
3	MIS	Mic	is	10	10
3	RC	Ryt	ctrl	10	7
3	REN	Ryt	en	10	7
3	RES	Ryt	es	10	9
3	RIN	Ryt	in	10	8
3	RIS	Ryt	is	10	10

Seed sown	9/05/2017
Thinned to 10	16/07/2017
Survival	11/03/2018

Aus: Austrostipa scabra
Mic: Microlaena stipoides
Ryt: Rytidosperma geniculatum

ctrl: untreated control
en: encrusted witouh SA
es: encrusted with SA
in: imbibed witouh SA
is: imbibved with SA

BOX EXPERIMENT					
Rep	Code	Species	Trt	Initial	Survived w45
4	AC	Aus	ctrl	10	4
4	AEN	Aus	en	10	9
4	AES	Aus	es	10	10
4	AIN	Aus	in	10	7
4	AIS	Aus	is	10	7
4	MC	Mic	ctrl	10	7
4	MEN	Mic	en	10	10
4	MES	Mic	es	10	10
4	MIN	Mic	in	10	8
4	MIS	Mic	is	10	10
4	RC	Ryt	ctrl	10	10
4	REN	Ryt	en	10	8
4	RES	Ryt	es	10	10
4	RIN	Ryt	in	10	9
4	RIS	Ryt	is	10	9

Seed sown	9/05/2017
Thinned to 10	16/07/2017
Survival	11/03/2018

Aus: Austrostipa scabra
Mic: Microlaena stipoides
Ryt: Rytidosperma geniculatum

ctrl: untreated control
en: encrusted witouh SA
es: encrusted with SA
in: imbibed witouh SA
is: imbibved with SA

SURVIVAL
AT 45 WEEKS

Code	Species	Trt	Avg	St.err
AC	Aus	ctrl	82.50%	12.44%
AEN	Aus	en	95.00%	2.50%
AES	Aus	es	97.50%	2.17%
AIN	Aus	in	80.00%	6.12%
AIS	Aus	is	90.00%	6.12%
MC	Mic	ctrl	82.50%	6.50%
MEN	Mic	en	100.00%	0.00%
MES	Mic	es	100.00%	0.00%
MIN	Mic	in	85.00%	8.29%
MIS	Mic	is	100.00%	0.00%
RC	Ryt	ctrl	85.00%	5.59%
REN	Ryt	en	77.50%	7.40%
RES	Ryt	es	97.50%	2.17%
RIN	Ryt	in	82.50%	7.40%
RIS	Ryt	is	92.50%	2.17%

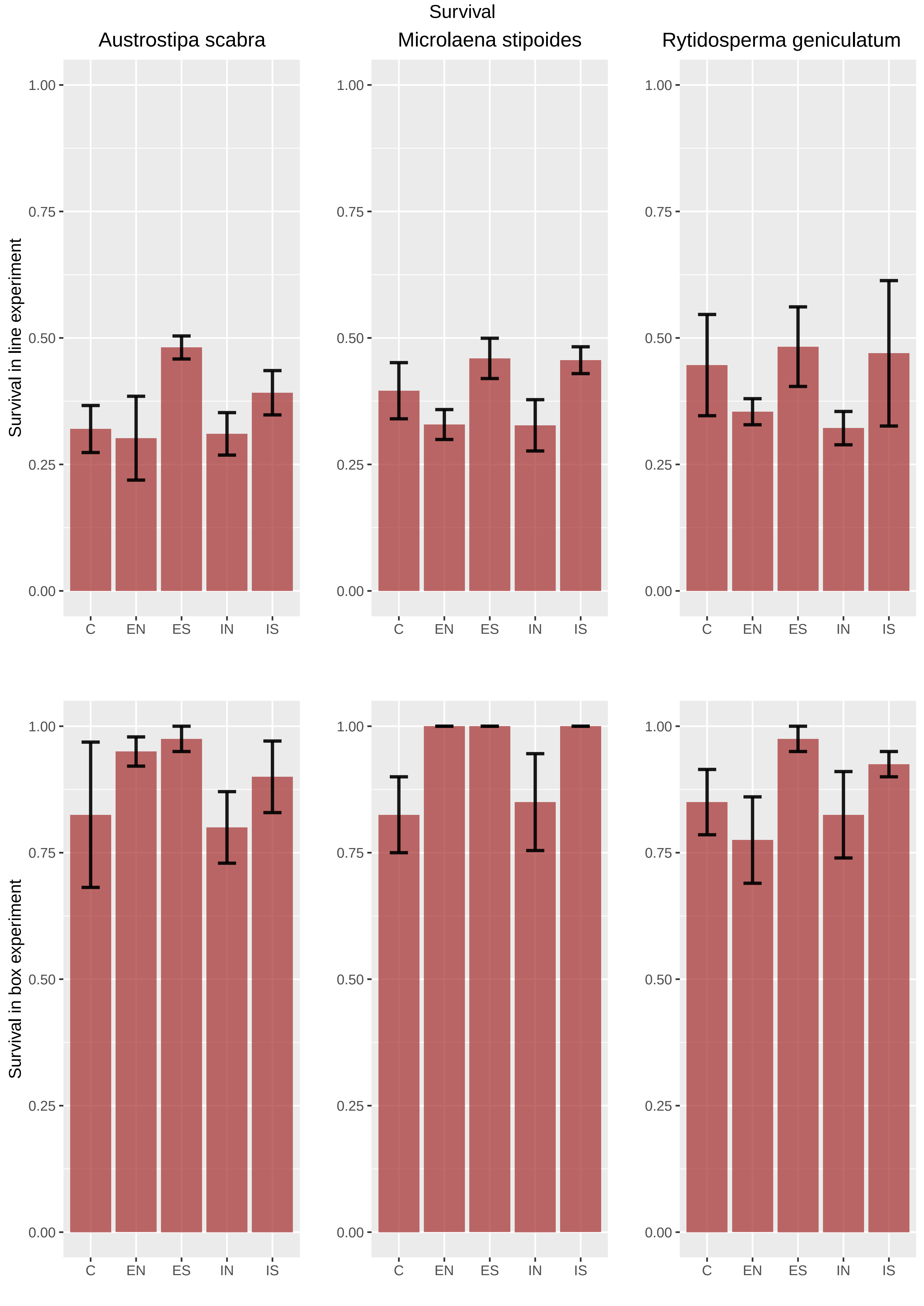

Survival
Austrostipa scabra
Microlaena stipoides
Rytidosperma geniculatum
Survival in line experiment
Survival in box experiment
1.00
0.75
0.50
0.25
0.00
C
EN
ES
IN
IS

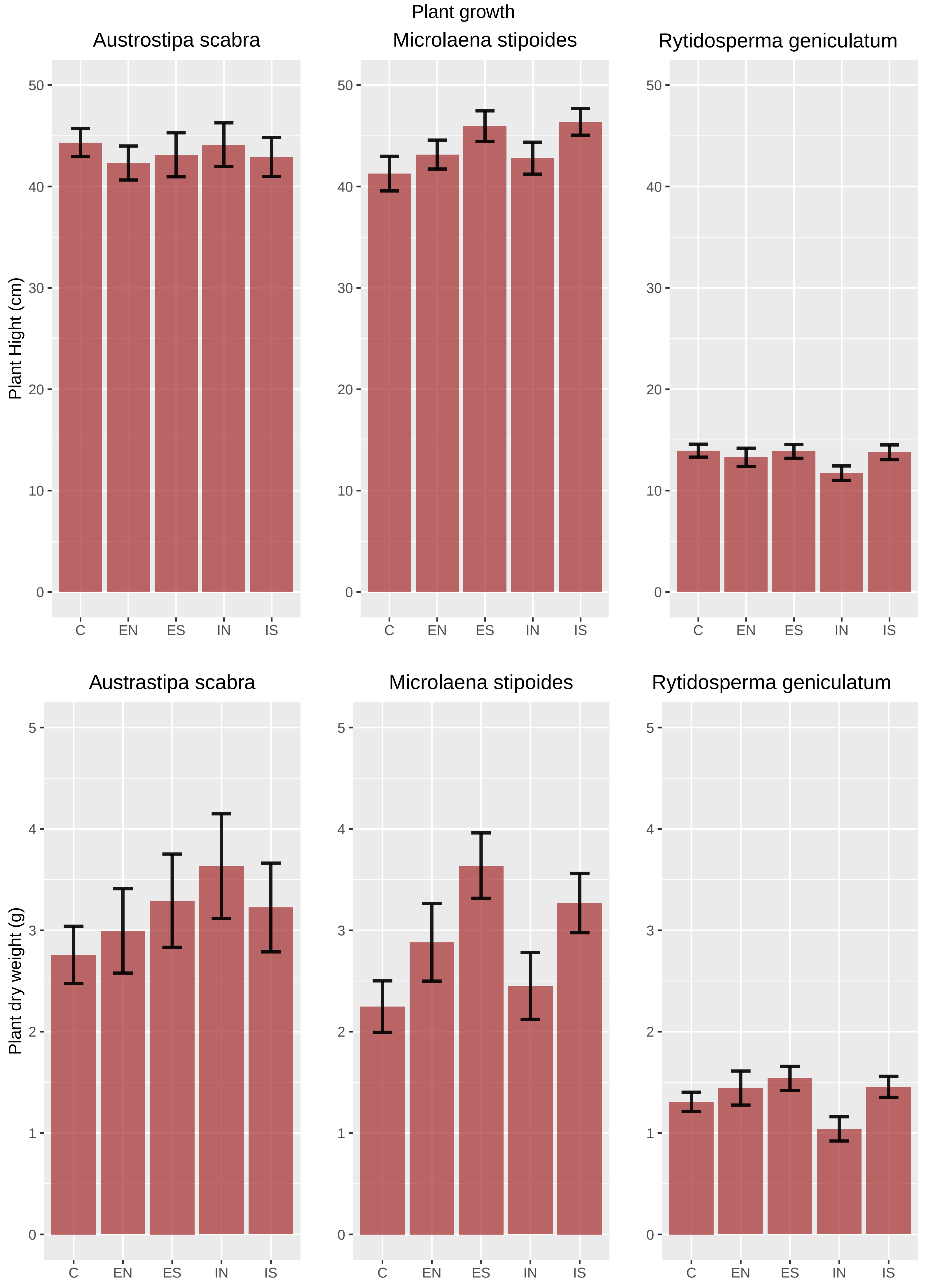

Plant growth
Austrostipa scabra
Microlaena stipoides
Rytidosperma geniculatum
Plant Hight (cm)
0
10
20
30
40
50
C
EN
ES
IN
IS
Austrastipa scabra
Microlaena stipoides
Rytidosperma geniculatum
Plant dry weight (g)
0
1
2
3
4
5
