# Supplementary material for: Seed encrusting with salicylic acid: A novel approach to improve establishment of grass species in ecological restoration
Source: PLoS One. 2021 Jun 9;16(6):e0242035. doi: 10.1371/journal.pone.0242035 (PMC8189473; doi:10.1371/journal.pone.0242035)
Supplement: Attachment — Submitted filename: Response to Reviewers 2.docx [file pone.0242035.s008.docx]

PONE-D-20-33303R1
Seed encrusting with salicylic acid: a novel approach to improve establishment of grass species in ecological restoration
PLOS ONE

Dear editor and reviewers

Thank you for providing further review and feedback on the manuscript. We have addressed the reviewers’ comments, noting the editor’s support for the opinion of Reviewer 2, with responses in red. I hope that this response and revision of the manuscript satisfactorily address the reviewers’ concerns.

Kind regards

Simone Pedrini

Review Comments to the Author

Reviewer #1: Pedrini et al have provided a revised version of their manuscript based off back from two reviewers. One of these reviews was provided by me.
After re-reading the manuscript I still do not feel the two major issues have been addressed suitably for progressing this paper to publication.

First, even though the authors have provided a map of the field design (“S1_FieldExperimentLayout) and some adjustment to the description in the methods and review responses (“limited space that was fenced”), the authors have not addressed the pseudo-replication of this study. The “Line”, “Bags”, and “Box” treatment appear to be replicated at least 4 x times within the trial area, however, this is not replicated at the site level. This lack of site level replication needs to be addressed as it is a flaw in the experimental design that could influence the findings and overall conclusions. What is to be consider pseudo-replication is still a controversial issue with useful commentary relevant to the biological sciences outlined in [*Davies GM, Gray A (2015) Don’t let spurious accusations of pseudo-replication limit our ability to learn from natural experiments (and other messy kinds of ecological monitoring). Ecology and Evolution 5:5295–5304*](https://onlinelibrary.wiley.com/doi/full/10.1002/ece3.1782). The trial area in the study site is a unique region within a high-profile national park where the study species naturally occur. As outlined in Davies and Gray (2015) there are particular reasons when it is permissible to conduct experiments within one site location when constraints such as ‘avoidance of clearing other areas in a national park’ are important concerns. The trials were replicated albeit within a fenced area

Second, and after the authors attempted to clarify the use of treatments and the use of acronyms, it is now apparent that the way treatments were ‘lumped’ and statistically analysed is problematic. For instance, the authors lump salicylic acid (both soaked “IS” and encrusted “ES” = IS+ES) treatments together along with the non-salicylic acid treatment (IN+EN) and carry out one level of analysis compared against un-treated control seeds. These two treatments within each lumping are not independent and I fail to see how you can validly lump them together for analysis and then separate them again later for a second post hoc assessment as independent treatments (if significance was found in the first step). Further, at the very basic level of tested seed treatments it is very odd to group something like seeds that have been soaked in SA with seeds that have been encrusted with SA – they are two different approaches and it makes no scientific sense to group them. Extended commentary on this flaw is provided below but a fear there is a lack of treatment independence that makes any findings or conclusions of this study inaccurate. Until this data is presented and analysed in a different manner, I do not feel this paper is suitable for publication. This comment is addressed as below.

Line by line comments provided below for other minor comments/thoughts (note, no detailed review was provided from the discussion down as the methods/results must be fixed first):
L 32-33 - would suggest swapping seedling growth to before plant survival Done

L 33 - Suggest spell out SA at the start of a sentence Done

L 41 - where are the figures to support the ‘cost-effective’ nature of this comment. Good point. A cost benefit analysis was not performed in this study. The sentence has been rephrased as follow: *Overall, adjustment of seeding rate according to expected emergence combined with the use of salicylic acid via coating could improve seed use efficiency in seed-based restoration.*

L 60 – Not sure if this sentence quite works in the way it is presented. Seed deployment is highly efficient in many degraded systems. For instance, native seed is spread very efficiently across the western USA using traditional sowing practices such as broad acre drilling. So, I would argue that its not ‘deployment’ but more so the conversion of this seed to an established plant that needs improvement in efficiency (e.g. "efficiency of seed deployment" is not quite the problem). My experience in Europe and Australia is that delivery is still a major bottleneck, especially when dealing with diverse seed mixes with complex morphological seed structures (such as the grasses in this study). However, I agree that seed deployment alone is not the main issue. I have therefore rephrased the sentence as follows: *Given the high cost and often highly limited availability of native seed, improving the efficiency of seed use (deployment, germinaition, and plant establishment) is crucial if ecological restoration is to be delivered at the expected landscape scale.*

L 72 - Unusual for acronyms to be used to start a sentence just like numerals. Suggest to spell out throughout paper. Done

Line 110 – Use of caryopsis appears incorrect. From the wording I would assume this would be the florets being immersed. Yes, caryopsis in line 113 was incorrect, and has been replaced with floret: *…with complete immersion of the floret in a 50% sulphuric acid solution…*

L 134 – “in a 15cm” Done

L 160 - desiccation of what? Filter paper, added

L 161 - sufficient hydration of what? Filter paper, added. *Germination tests were performed in Petri dishes lined with two filter papers moistened with 14 ml water or Polyethylene Glycol (PEG) solution and placed in sealed plastic bags to reduce filter paper desiccation. Each week, 2 ml of water or PEG solution was added to ensure sufficent hydration of the filter paper.*

L 164 - The use of 'this value' here is unclear as is infers ‘one value’ and then a range is referred to for water available - please clarify language.
Do you mean "this lab-based water potential gradient" resembles the range of water available recorded in the field during the winter months. If this is what is meant, this is not reflected in the field data as volumetric water content appears to not exceed 0.17 or 17%. Also the field data over your winter months doesn’t seem to represents anything close to 'field capacity’ with a rough conversion of of this data to be >0.2MPA. So, the usefulness of, ab comparisons to field results would be questionable. I agree with this point. The field site never reached field capacity. I have rephrased the sentence to acknowledge this: *The values at reduced water potential resembles the range of water availability recorded in the field during the winter months. (S2 Appendix, Soil Water retention curve).*

L 216-217 - These are not independent treatments and I cannot not see how this is a valid lumping of independent variables. See comments further down.
L 249 (whole section) - From my first review I appreciate that the authors have attempted to clarify the use of the treatment acronyms in the worded text and figures. In this revised version I now worry that the study design and the way it is presented is problematic.
It now appears, that at least in this section, the treatments have been lumped together in the data presentation and statistical assessment.
This is a major problem for the paper. Unless there is a new way to lump two "independent" treatments together (e.g. in this instance IS & ES 'lumped' and IN & EN 'lumped') I cannot see how you can conclude the success of any of these treatment over another.
For what I can tell from your statistics methods (ca. Line 216) you have analyzed SA 'lumped' together and the two non-SA treatments 'lumped' together against the un-treated CTRL. Then if statistically significant effects were found you then assess the ‘within’ SA or non-SA comparisons (here for instance whether ES was different to IS). The first lumping is just not independent (i.e. 4 treatments lumped into 2 groups) and I struggle to see how this a valid approach to take the next ‘within’ post hoc step (compare single independent treatment informed from the first lumping).
To me this section needs a solid re-write as there are two independent lines of investigation that needs to be presented separately or in a different manner.
In one set there could be a clear presentation of CTRL v IN v IS and in the second CTRL v EN v ES. If SA provides the hypothesized desired benefits, then you can conclude its treatment benefit over the CTRL and whether the SA treatment worked over the soaking in water or coating with no-SA.
An alternative is to provide all treatments in the column graphs of Figure 4 (e.g. five columns with CTRL, IN IS EN ES) and compare each of these independent treatments (IN IS EN ES) back to the untreated control (CTRL). Using something like a post hoc pairwise comparison such as Dunnett (using the ghlt function in the multcomp package in R which deals with many treatments being compared against one control) you can them map the effect size of these independent treatments against the focal control, and even to each other, to offer a valid independent conclusion.
As it stands, I think this analysis and data presentation is a problem and a major flaw.

L 281 (whole section) - My commentary is repeated here from the above comments surrounding the lumping and lack of Independence.

I understand the concern of the reviewer on the approach of lumping different treatments. When I was originally analysing the data, I have tested similar approaches to the one suggested by the reviewer by comparing each treatment to each other. However, this approach made the interpretation of the results harder and more confusing and did not allow direct testing of the hypotheses. The way the current analysis is performed, and the results presented, allows each hypothesis to be sequentially addressed (1. is SA beneficial? 2. Which method of delivering SA works better?). To address the reviewer’s concern on the validity of the results, I have provided bar chart graphs, with all five treatments (Ctrl, IN, IS, EN, ES) presented side by side, for all lab germination and field emergence (S4_GerminationEmergenceAnalysis.pdf) and bag germination, in line and box survival, and growth (S5_FieldExperimentsResults.pdf.) Those graphs confirm the same trends that are presented with the current analysis: SA does not improve germination and emergence, but provides benefits in terms of survival and, to a lesser extent, growth.

As this concern was not raised by the other reviewer and the editor, I would prefer keeping the analysis, results, and discussion as they currently are. If the editor or the other reviewer think it is better to change the statistical approach and rewrite the result and discussion accordingly, I will do so but it would not materially affect the outcomes with both approaches being valid.

------------------------------------

Reviewer #2: In general my comments have been addressed by the authors and I feel the manuscript will be ready for publication after some minor editing. There is still some grammatical issues in the manuscript. Some of examples are listed below

line 102, this phase is not clear "and utility as pasture." rephrased: “*were selected on the basis of their predominance in revegetation and restoration activities and their potential use for pasture in replacement of, mostly European, fodder crop.*

line 174, "at 1cm depth" change to "at a 1 cm depth" fixed

Line 241, need a period. Also on this line and through the manuscript you may want to add the word "the" before saying the treatment name. For example on this line it says ..."IN seeds showed no significant difference compared to CTRL." Perhaps you could say "IN seeds showed no significant difference compared to the CTRL." Additionally, in my opinion the use of the word "significant" is redundant. My preference would be to have the sentence say "IN seeds showed no difference compared to the CTRL." With that said, this is a style preference and the authors do not need to implement this change here and at other parts of the manuscript. Fixed here and throughout the document. The term significant was left when P value was provided and in cases that a difference was detected but was not statistically significant. E.G “*SA treatments generally showed a slight but non-significant improvement in final germination*”

Line 298, poor grammar Rephrased: *To evaluate treatment effects on plant growth plant height and above ground dry biomass were recorded.*

Line 303, for ease of reading would it may be better to keep the treatment order the same as the sentence above: sentence rephrased: *Dry above-ground biomass was also higher in SA treatment (IS+ES, 3.4 g ± 0.22g) compared to non-SA treatments (IN+EN, 2.7 g ± 0.25 g) and untreated controls (CTRL, 2.2 g ± 0.25 g)*

Line 309, Need period. Fixed

Lines 396 - 401, very long sentence with poor grammar Rephrased: “*A study evaluating the demographic processes that influence established of native species from seed in field trials, performed by James et al. (2011), identified the main bottleneck to successful seedling recruitment as the emergence phase (when germinated seeds failed to express surface emergence*

*0*”

Lines 397 - 401, consider expanding your discussion of the James et al. (2011) paper to talk about how your technology has application outside of the region this study was conducted. James et al. (2011) implemented a dormant seeding with seeds remaining in the soil under freezing soil conditions for 4-5 months prior to emergence. In cold desert regions similar to where James et al. (2011) conducted their study, high mortality can occur over the winter to the seeds and unemerged seedlings from freezing temperatures, pathogens, drought, and other stressors. Just as the SA treatment improved drought stress in your study it is probable that a SA seed treatment may mitigate limiting abiotic conditions associated with dormant winter seedings.

This is a good discussion point and was added at the end of the section: *The improved establishment of Australian grasses treated with SA suggest that a similar approach can be tested in different systems. For example, in cold desert restoration, where high seedling mortality occurs in the winter months due to a variety of biotic and abiotic stressors such as freezing temperature, drought and pathogens, [12,58] SA application to seed prior to seeding could be a useful adjuvant to promote seedling survival.*

If you chose to add this topic to your discussion I would also cite James et al. (2019).
James, J. J., R. L. Sheley, E. A. Leger, P. B. Adler, S. P. Hardegree, E. S. Gornish, and M. J. Rinella. 2019. Increased soil temperature and decreased precipitation during early life stages constrain grass seedling recruitment in cold desert restoration. Journal of Applied Ecology 56:2609-2619.

Citation added
